# Health Impacts of the Green Revolution: Evidence from 600,000 births across the Developing World^☆^

**DOI:** 10.1016/j.jhealeco.2020.102373

**Published:** 2020-12

**Authors:** Jan von der Goltz, Aaditya Dar, Ram Fishman, Nathaniel D. Mueller, Prabhat Barnwal, Gordon C. McCord

**Affiliations:** a World Bank; b Economics and Public Policy Area, Indian School of Business, Hyderabad; c Department of Public Policy, Tel Aviv University; d Department of Ecosystem Science and Sustainability & Department of Soil and Crop Sciences, Colorado State University; e Department of Economics, Michigan State University; f School of Global Policy and Strategy, University of California, San Diego

**Keywords:** Agricultural technology, Modern seed varieties, Green Revolution, Infant mortality

## Abstract

What is the contribution of the ‘Green Revolution’ to improvements in child health during the $20^{\mathrm{th}}$ century? We provide global scale estimates of this relationship by constructing a novel, spatially-precise indicator of modern crop variety (MV) diffusion and leveraging child-level data from over 600,000 children across 21,604 sampling locations in 37 developing countries between 1961–2000. Results indicate that the diffusion of MVs reduced infant mortality by 2.4–5.3 percentage points (from a baseline of 18%), with stronger effects for male infants and among poor households. The sizable contribution of agricultural technology to improved welfare should inform global food and development policy.

## Highlights

• The ‘Green Revolution’ had large and positive effects on child health
• One standard deviation increase in modern variety seed diffusion led to a 1.3 percentage point decline in infant mortality
• The reduction in infant mortality is larger for male infants and among poorer households

## 1 Introduction

The Green Revolution is one of the most important technological transformations of the $20^{\mathrm{th}}$ century. In the course of the past 60 years, modern crop varieties (MVs) of staple crops, developed by dozens of national agriculture programs with the support of international agricultural research centers, spread across the developing world. Along with complementary inputs, MVs have been a key driver of dramatic increases in crop yields (Evenson and Gollin, 2003a), which vastly improved food supplies and may have triggered broader economic development and structural transformation (McArthur and Sachs, 2019, Dall Schmidt et al., 2018, McArthur and McCord, 2017, Bustos et al., 2016, Nunn and Qian, 2011). Africa's relative lack of a green revolution is often cited as a key reason for why the region has not yet experienced greater long-term economic success (Diao et al., 2010, Byerlee et al., 2009).

Despite widely-held agreement about the contribution of the Green Revolution to global aggregate food supply, micro-level evidence on how the diffusion of MVs affected human welfare, particularly health, remains remarkably scarce (Masters et al., 2014). Credible evidence on this question could provide important insights on the linkage between increased economic productivity and health, which remains imperfectly understood particularly for low income households. It also contributes to the debate about the relative role of increased incomes (as opposed to public health advances) to the $20^{\mathrm{th}}$ century's dramatic decline in infant mortality (Cutler et al., 2006, Pritchett and Summers, 1996, Preston, 1980, Ruhm, 2000), particularly for rural populations in developing countries where incomes are strongly tied to agricultural productivity growth (Bhalotra, 2010). In addition, this evidence adds to the renewed discussion about the degree to which agricultural productivity gains can improve human health in developing countries, be it through nutritional or income channels. The topic is of great interest to development policy, but several reviews lament the shortage of credible evidence on the question (Webb, 2013).^1^

In this paper, we estimate the localized (sub-national) impact of MV diffusion during the Green Revolution on infant mortality (IM) at a scale that has not been attempted to date. Our analysis makes use of spatially precise survey data from the Demographic and Health Surveys (DHS) on the mortality of about 600,000 children born between 1961 and 2000 across 21,604 rural sampling locations in 37 countries. In order to overcome the unavailability of large scale sub-national data on MV diffusion, we employ a novel approach that exploits the spatial variation in local crop-share and the temporal variation in national-level MV adoption to construct a spatially explicit, time-varying modern variety diffusion index (MVDI) at 5 arc minute resolution (around 10 km at the equator). We then estimate the within-country association between this local prediction of MV diffusion and IM using a difference-in-differences approach that controls for DHS sampling cluster fixed effects and flexible country-specific time trends. Our identification strategy therefore exploits sub-national variation in MV diffusion resulting from the interaction of the local crop mix in each country and differences in MV diffusion rates across crops in the same country, which largely reflects variation in international research performance across crops. We subject this model to a wide range of demanding robustness tests.

Localized increases in crop yields resulting from MV adoption can lead to local health improvements through both nutritional and income channels, and may depend on whether a household is a net food seller or buyer (Aksoy and Isik-Dikmelik, 2008). For food insecure subsistence farmers, higher yields can directly lead to increased food intake. For farmers who are net food sellers, income may or may not increase, depending on how far prices decline as total production increases. Non-farming households may also benefit if increased production reduces local food prices in imperfectly connected markets, through higher consumption of either food or other (potentially health-enhancing) goods. On the other hand, the Green Revolution has also been criticized for potentially failing to reach the lowest income households, focusing entirely on caloric output and for its environmental impacts (Pingali, 2012). There is evidence that the intensive use of agro-chemical inputs that typically accompanies MV adoption has adverse environmental and health effects, including on IM (Brainerd and Menon, 2014, Dias et al., 2019). The net effect on health and IM is therefore unclear and a topic of ongoing debate, which, in turn, has important implications for current policy debates on the merit of continued investments in staple crop improvements and the diffusion of modern varieties, particularly in sub-Saharan Africa.

We find a large and statistically significant impact of MV adoption on infant mortality. Results indicate that the diffusion of MVs reduced infant mortality by 2.4–5.3 percentage points (for comparison, the beginning-of-period infant mortality was 18% in 1961-65, and decreased to 8% by the end of the sample period in 1995-2000). The effect is stronger for male infants. Results are robust to various alternative definitions of the MVDI as well as controlling for indicators of other drivers of IM decline such as maternal education levels or access to public health, controlling for predictors of localized economic growth such as access to trade, removing crop-specific trends that could potentially be driving the association, and limiting the comparison to siblings.

The paper makes several contributions. First, it quantifies a key relationship between two major transformations in the last 60 years of human history – agricultural productivity increases and health improvements – at a spatial scale and precision not previously possible. Studying this relationship at global scale is important because MVs diffused at unequal rates across regions (with Asia at one extreme and sub-Saharan Africa at the other) and because local covariates might affect this relationship in ways that shape the welfare gains of MVs. Second, the paper offers a methodological contribution in the construction of the MVDI by exploiting spatially explicit data on historical crop shares with time-varying data on agricultural technology advances in order to generate a local measure of MV diffusion. This geospatial method for constructing sub-national data on MV diffusion has useful analogues and applications in other empirical contexts. For example, Dias et al. (2019) construct a municipality-level variable for herbicide usage in Soy production in Brazil. Third, the results are of core interest to policy. If the average MV diffusion rate in sub-Saharan Africa were to increase to South Asian levels (around 60%), our global estimates imply that IM in SSA would decline by around 31% from the 2010 infant mortality rate of 65 per 1,000 live births (World Bank, 2015). This reduction would be comparable to the benefits of reducing particulate matter pollution in SSA to WHO recommended levels and of achieving universal coverage of several public health interventions (Heft-Neal et al., 2018). Finally, the observation that effects of MV diffusion on child health vary significantly by child sex contributes to the existing literature on sex-specific health outcomes for children.

Previous research has shown that IM responds to aggregate income shocks (Baird et al., 2011). Multiple studies have investigated the impacts of the Green Revolution on agricultural and economic outcomes using country-level data (e.g., Evenson and Gollin (2003a), Walker and Alwang (2015)). Country-level analyses are typically prone to limitations when they involve variables that display substantial sub-national heterogeneity, and the relatively small sample size limits statistical power.^2^ Moreover, any association is difficult to interpret causally, since it could be driven by latent aspects of overall economic development. For example, countries experiencing faster economic growth in non-agricultural sectors might be better placed to invest in both agriculture (perhaps through subsidizing MVs) and public health. Two recent advances in this regard are offered by Gollin et al. (2018) and Moscona (2019), who employ quasi-experimental research designs that exploit agro-ecological spatial variation in MV suitability and time variation in development of new MV technologies for various crops. Gollin et al. (2018) find large impacts on income and mortality, whereas Moscona (2019) do not find evidence for positive impacts on national income.^3^

The lack of availability of sub-national data on MV adoption has made it difficult to go beyond cross-country analyses, at least at large scales.^4^ An exception is the small but burgeoning literature that exploits sub-national variation within countries (Sekhri and Shastry, 2019, Bharadwaj et al., 2020, Moscona, 2019). Providing a long-term perspective, Sekhri and Shastry (2019) shows that an increase in total calories and fat intake due to the Green Revolution led to adverse adult health outcomes in India. Moscona (2019) finds MV adoption stimulated the agricultural sector but shrank manufacturing. Bharadwaj et al. (2020), in a study most related to ours, employ a district-level difference-in-differences strategy and find that MV adoption reduced infant mortality in India. Even though our data covers a large number of developing countries (including India) and our analysis pursues a very different empirical strategy, our estimates on the impact of MV adoption on infant mortality are similar to those found by Bharadwaj et al. (2020). In that sense, the two studies validate each other's internal and external validity in a manner seldom possible. This makes it possible to generalize the conclusions beyond the Indian context, which is particularly relevant for sub-Saharan Africa, where the diffusion of MVs and other modern agricultural technologies is lagging behind other developing regions. The remainder of the paper is structured as follows: Section 2 discusses data and empirical methodology, Section 3 describes results, and Section 4 concludes.

## 2 Data and Empirical Strategy

### 2.1 Data Sources

#### 2.1.1 Demographic and Health Surveys

Our main outcome variable, infant mortality, is measured through Demographic and Health Surveys (DHS), which are the only high-quality, spatially-referenced, and internationally-comparable household surveys that provide detailed information on health at the individual level. Pooled DHS survey data have been used for numerous studies on the impacts of pollution and income shocks on child health (von der Goltz and Barnwal, 2019, Heft-Neal et al., 2018, Baird et al., 2011). We compiled DHS data for developing countries in the following regions: sub-Saharan Africa, North Africa, Latin America, South East Asia and South Asia. Each DHS surveyed women of ages 15-49 regarding their fertility history, generating records for about three million children. In our preferred specification, we restrict the data to rural areas and to mothers who have never migrated, since we are assigning the exposure of each child to MV diffusion according to their location.

We focus on children born between 1961 and 2000, given the available data on MV diffusion. We use the recalled birth dates, survival at survey time, and date of death of her children (along with other basic information on the child's birth) from the birth history of up to 20 children born to the respondent. Data are then transformed into individual records for each child born, and a binary infant mortality variable records whether the child died before she reached twelve months of age. Figure A1 shows the distribution of child births over time in our sample.

While it is very unlikely that a mother will fail to recall the birth of her child, recall bias (regarding the timing and omission of distant births) may be a potential concern for studies using this data. In our case, since the MV diffusion data is reported only every five years, inaccuracies in reporting the timing of births are likely a lesser concern. Additionally, the errors in recall would have to be correlated with crop share variation across the country in order to bias our estimate of the effect of MVDI on IM. Any ubiquitous problems with recall would be absorbed by the flexible detrending at the country level.

**Fig. 1:**
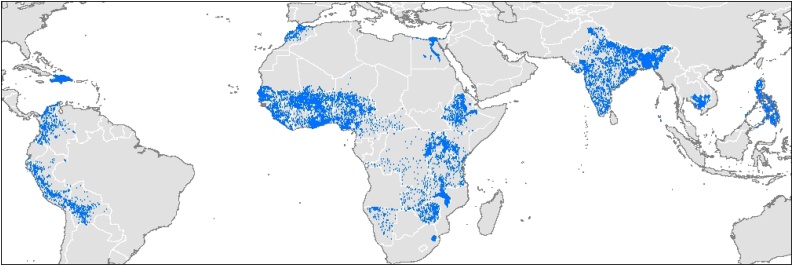
Spatial distribution of infant mortality data from the Demographic and Health Surveys. Note: Dots represent the locations of Demographic and Health Survey clusters used in the analysis (N = 21,604 clusters across 37 developing countries).

The resulting sample (once matched with the MV diffusion data) includes 21,604 DHS sampling clusters^5^ in 966 administrative regions spread across 37 countries. The DHS are geo-referenced to roughly within 5 km in rural areas, which can be spatially merged with crop distribution data allowing for an analysis at high spatial resolution. Using the georeferenced DHS data (as opposed to DHS surveys geolocated only to a district or other larger administrative unit) is important because of significant spatial variation in both IM and crop mix, and because exploiting the rich subnational variation is key to explaining most spatial variation in child mortality (Burke et al., 2016). The DHS clusters in our study are mapped in Figure 1.

Further, we extract data on covariates such as mother's age at birth, mother's literacy and occupation, and socioeconomic characteristics of households from various DHS modules, which we match with the child-level information on infant mortality. We use the data on socioeconomic characteristics of households to construct a poverty score using standard principal component analysis method, which serves as a proxy measure of latent wealth characteristics of rural households. The score includes socioeconomic household traits like education, marriage status, type of floor, roof and wall, ownership of agricultural assets such as livestock, land and animal cart and follows the approach in von der Goltz and Barnwal (2019). The score is created only for rural areas and the scale is reversed so that higher values indicate fewer assets. It is important to clarify that some of the data (such as mother's education, occupation and poverty score) are available only at the time of survey, not at the time of each child's birth.

In addition to data on infant mortality, the DHS also collects anthropometric measures that can be used to measure malnutrition among children. We use four such measures as a secondary outcome in our analysis: severe stunting (defined as being more than three standard deviations below the age-specific mean of height-for-age), severe wasting (three standard deviations below median weight-for-height), severe underweight (three standard deviations below median weight-for-age), and low birth weight (child's weight at birth less than 2,500 grams). Unlike the birth histories which are used to construct infant mortality, these data are available only for recent births in relation to the year of survey.

Finally, since improvements in public health are an obvious driver of IM change, we leverage the richness of the DHS to create proxies such as access to health care (women reporting that distance was not an obstacle in the use of medical care), antenatal care visit (number of antenatal visits reported by women), institutional delivery (children who were reported to have been delivered in any kind of health facility), breastfeeding (women who reported to be breastfeeding at the time of survey), and vaccination rates (children who received any vaccination for BCG, TB, DPT, Polio, Measles, etc.).

Table 1 provides summary statistics, and Table A2 lists the countries in the main sample, the number of DHS rounds available, and the number of male and female children in the sample.

**Table 1:** Summary Statistics

	Mean	Std.Dev.	Obs
Year	1989.02	7.71	612,031
*Outcome*			
Infant Mortality: All	0.10	0.30	612,031
Infant Mortality: Girls	0.10	0.29	297,872
Infant Mortality: Boys	0.11	0.31	314,159
*Treatment(% crop area planted to MVs)*			
EarthStat (circa 2000)	0.14	0.17	597,251
SPAM (circa 2000)	0.13	0.18	577,101
EarthStat (1961-65)	0.18	0.21	581,494
*Controls*			
Sex ratio	0.51	0.50	612,031
Mother's age at birth	24.27	6.16	612,031
Mother is not literate	0.69	0.46	467,776
Mother is wage worker in agriculture	0.09	0.28	571,766
Rural poverty index	0.01	0.59	591,109

Note: Data come from rural clusters in 37 countries where DHS geocoded data and MV data are available. The number of observations (N=612,031) refer to the union of estimating samples (EarthStat 2000, EarthStat 1961–65 and SPAM 2000).

#### 2.1.2 Modern Variety Diffusion Data

Modern varieties are defined as the crop genotypes developed by International and National agricultural research centers (IARCs and NARCs) that spread throughout the developing world beginning with the Green Revolution in the early 1960s. Common breeding objectives for modern varieties are high yield potential; resistance to stress, pests, and disease; and improved quality of the harvested material (Byerlee et al., 2009). We utilize a historical, country-level dataset on MV diffusion from a study commissioned by the Impact Assessment and Evaluation Group of the CGIAR's Technical Advisory Committee, also summarized by Evenson and Gollin (2003a). The dataset (referred to as EGMV from here onwards) was assembled utilizing country-specific MV introduction and diffusion data to create a complete time series of MV adoption rates in 5-year intervals for 11 major crops (wheat, maize, rice, barley, pearl millet, sorghum, cassava, potato, groundnut, beans, and lentils) in 90 countries between 1960 and 2000. The original data were constructed from expert opinion surveys and (for some crops) administrative records and surveys. MV adoption rates denote the fraction of crop area planted in modern varieties relative to the total area planted in both modern and traditional varieties. Note that the crops for which MV data are available are important staple crops in terms of caloric intake, and cover 60% of cropland in our sample locations on average. Our analysis only uses data for the 37 countries for which geo-referenced DHS data are available.

#### 2.1.3 Global Crop Maps

We employ three global crop datasets, each providing spatially explicit data on localized crop mixes. These datasets report the area cultivated by crop in every location of the world, and are used to construct predicted local MV diffusion rates. All three crop datasets provide global maps at a five arc-minute resolution (around 10 km grid cells at the equator), but they differ in terms of crops covered, data sources, and methodology. The three crop maps allow for constructing three versions of the MVDI, which we use to test the robustness of the main results.

The first crop dataset is from EarthStat.^6^ Monfreda et al. (2008) reports harvested area data circa 2000 (1997-2003) for 175 crops, of which 11 are relevant to our analysis due to the availability of EGMV data. This dataset uses agricultural census and survey information to distribute crop harvested area across physical cropland areas, which are determined from remote sensing and agricultural census and survey information.

The second crop dataset is the Spatial Production Allocation Model (SPAM). Similar to the EarthStat data, the SPAM maps are based on a collection of agricultural census and survey data, but the disaggregation to the grid cell of crop harvested area is based on a modeling approach that includes information on total cropland areas, biophysical crop suitability assessments, population density, and crop prices (You et al., 2014). SPAM includes crop harvested area data for 10 crops circa 2000 for which EGMV data is also available.

The third crop dataset is also from EarthStat, but reports yearly historical harvested area data from 1961–2008 (Ray et al., 2012), thus including years before the onset of the Green Revolution. The advantage of using plausibly exogenous crop shares comes at the cost of sacrificing crop coverage, as EarthStat 1961-65 only covers three major cereal crops for which EGMV data is available: maize, wheat, and rice.^7^ The spatial and temporal frequency of the source data differs by country, and is not as complete as EarthStat 2000. When historical subnational data is not available in a given country, the harvested area estimates are determined from the circa 2000 crop distribution data and historical national-level data.

### 2.2 Construction of Modern Variety Diffusion Indicator

To overcome the fact that subnational data on MV diffusion over time is extremely sparse in developing countries, we construct a high-resolution prediction of local MV diffusion which we refer to as the Modern Variety Diffusion Indicator (MVDI). The MVDI is constructed by combining high-resolution global crop maps with country-level, crop-specific data on MV diffusion over time (1960-2000) available from Evenson and Gollin (2003b). Variation in the MVDI therefore combines fine spatial variation in cropping patterns with crop-specific temporal variation in the diffusion of MVs, which partly results from differences in international agricultural research priorities and breakthroughs across crops during the course of the Green Revolution.

The onset of the Green Revolution and its subsequent patterns of diffusion can be considered exogenous to specific countries. International crop research programs led to improved varieties, which were then localized by national agricultural research centers. During 1960-2000, MVs diffused across the developing world in stages, largely dictated by technological advances at the IARCs for different crops and different agroecological zones. Early successes in the 1960s benefited wheat and rice varieties, in part because technologies available for these crops in developed countries could be easily transferred. Breeding programs for many other crops had no such earlier science to rely on, and thus modern varieties for crops such as sorghum and millet only became available significantly later (in the 1980s). The arrival of MVs at a given location and time, therefore, was determined to an important extent by the scientific advances in the IARCs, the location's agroecological suitability for different crops, and how much additional breeding would have to be done by NARCs (Evenson and Gollin, 2003a).

The MVDI is constructed in each grid cell and 5-year time step as the weighted average of crops’ MV diffusion rate in that year (reported at the country level by Evenson and Gollin (2003b)), where the weights represent the relative share of cropped area in that grid cell devoted to that crop. To test whether the results are driven by particular features of a single crop dataset, we develop and analyze three variants of the MVDI based on three distinct global crop map datasets as mentioned earlier (Monfreda et al., 2008, You et al., 2014, Ray et al., 2012). The MVDI is constructed as follows:

(1) $${\mathrm{MVDI}}_{\mathrm{vct}}=\frac{\sum_{j=1}^{J}\left({\mathrm{CropArea}}_{\mathrm{jvc}}\times {\mathrm{EGMVArea}}_{\mathrm{jct}}\right)}{\sum_{j=1}^{J}\left({\mathrm{CropArea}}_{\mathrm{jvc}}\right)}$$

where, v is a location (DHS cluster) in country c and t is the period of observation. ${\mathrm{EGMVArea}}_{\mathrm{jct}}$ is the share of area cultivated with crop j that is planted with MVs in country c at time t, and ${\mathrm{CropArea}}_{\mathrm{jvc}}$ is the area cultivated with crop j in location v, as reported in the global crop maps (which are time invariant) mentioned above. The summation is conducted over all crops covered by the crop map in question: for the EarthStat circa 2000 data (Monfreda et al., 2008), J = 11 (barley, bean, cassava, groundnut, lentil, maize, millet, potato, rice, sorghum, wheat); for the SPAM dataset (You et al., 2014), J = 10 (barley, bean, cassava, groundnut, maize, millet, potato, rice, sorghum, wheat); and for the historical EarthStat data (Ray et al., 2012), J = 3 (maize, rice, wheat).

**Fig. 2:**
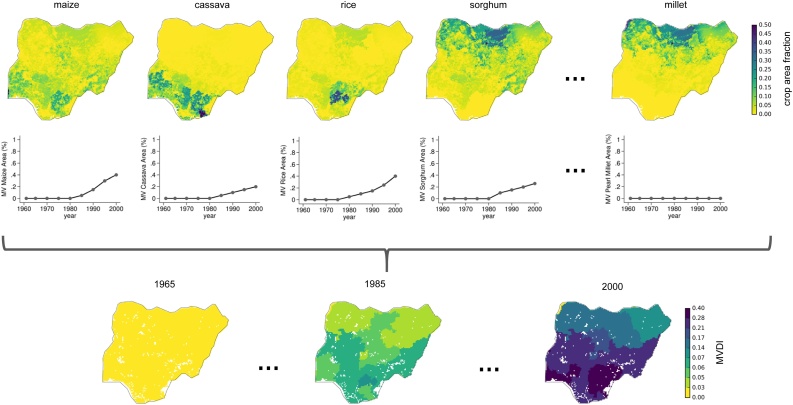
Constructing the modern crop variety diffusion indicator (MVDI) for Nigeria. Note: In each location, country-level crop specific modern variety diffusion data Evenson and Gollin (2003a) is combined using the local crop mix, obtained from global, spatially precise crop map datasets. MVDI represents the fraction of local crop harvested area allocated to a modern variety.

**Fig. 3:**
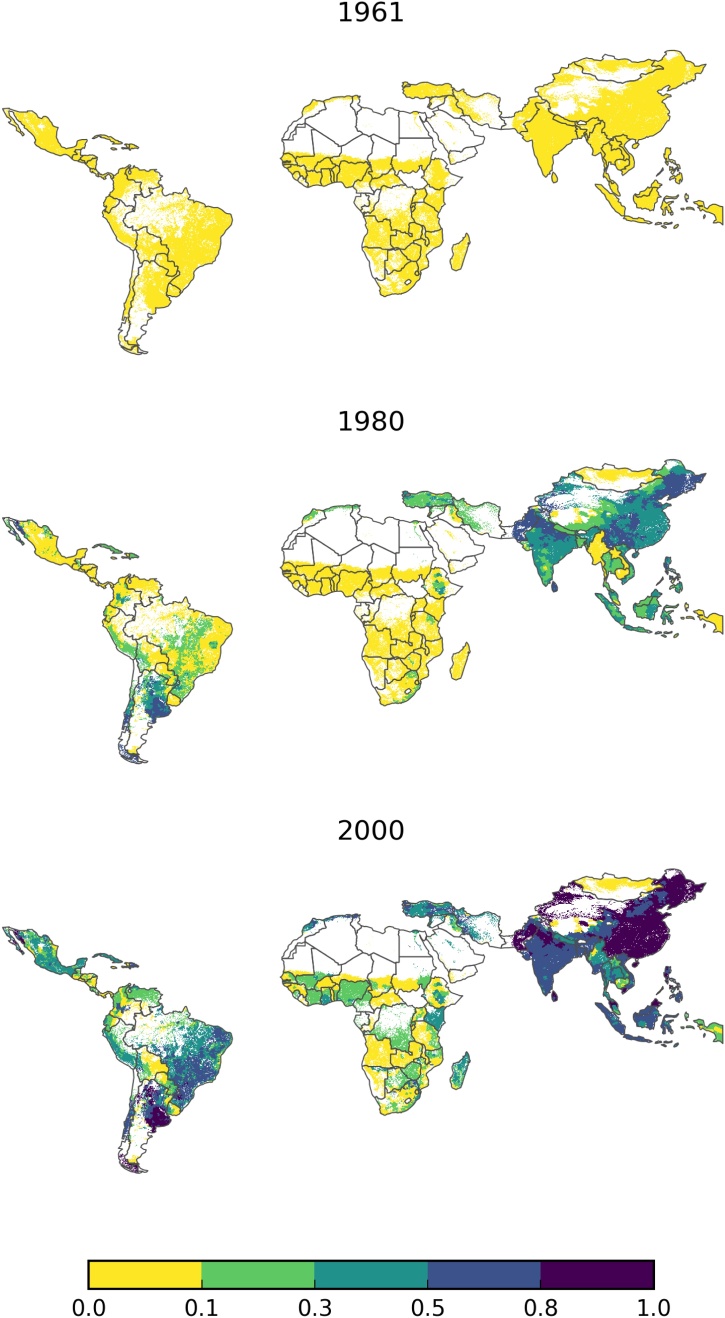
Historical changes in the Modern Variety Diffusion Indicator (MVDI) using EarthStat 2000 crop areas at three points of time. Note: Here we visualize changes in MVDI for all 86 countries where MV data are available, but note that not all countries are included in the estimating sample because they lack georeferenced DHS data.

Figure 2 illustrates the construction of MVDI using one country as an example (Nigeria). We multiply the spatial distribution (top panel, this illustration uses data from Monfreda et al. (2008)) with the national-level MV diffusion (middle panel, data from Evenson and Gollin (2003a)) for all crops for which data is available (there are 11 such crops in EarthStat 2000 but only 5 are shown to conserve space). This approach generates a gridded map (bottom panel) of the diffusion of MVs in each year, weighted across all crops. For example, we can see that there is a relatively low rate of overall MV diffusion in northern Nigeria. This is because millet happens to be a dominant crop in that region and MVs for millet diffused late because IARCs did not produce relevant varieties until the 1980s (Evenson and Gollin, 2003a, Walker and Alwang, 2015). Figure A2 and A3 depict the construction of MVDI using SPAM data (You et al., 2014) and EarthStat 1961-1965 (Ray et al., 2012), respectively. Figure 3 shows the global distribution of the MVDI (using the EarthStat 2000 data) in 1965, 1985, and 2000.

Each of the three MVDI indicators has strengths and weaknesses. The variant that uses EarthStat’s 1961–1965 average cropped areas generates a more exogenous subnational prediction of local MV diffusion, since subsequent crop distributions might shift as a response to MV availability. On the other hand, using the Earthstat 2000 and SPAM data allows for more crops in the analysis, and sidesteps data quality issues in the construction of 1961–1965 cropland maps for countries lacking agricultural census data in earlier time periods^8^ .

The method used to construct the MVDI is similar in spirit to the Bartik-style shift-share approach (Bartik, 1991, Autor and Duggan, 2003, Autor et al., 2013).^9^ It takes a measure of temporal change at the aggregate level (national MV adoption, partly reflecting breakthroughs in international agriculture research), and considers variation in the degree to which different locations were exposed to this change given their relevant characteristics (crop mix). Our methodology to construct a grid-cell level and time-varying indicator for MV diffusion provides a unique approach to address the lack of sub-national level MV data in empirical research.

#### 2.2.1 MVDI as a downscaling of MV Diffusion

We use the MVDI as a prediction of the actual, but unobserved, localized rate of MV diffusion in the sample of DHS clusters. For the analysis to be interpreted correctly, these constructed proxies need to be highly correlated with actual diffusion rates. Since the local MV diffusion rates are not observed globally with comparable spatial precision, our ability to test the correlation of MVDI with actual local diffusion rate of MVs is limited. We can, however, perform two partial tests.

The MVDI is only a valid prediction if the fraction of area devoted to various crops remains well correlated over time. This can be verified by checking the correlation of grid cell crop areas between 1965 and in 2000 for countries in our sample with historical subnational census records (Ray et al., 2012). The correlation is 0.92 in the case of maize, 0.57 in the case of wheat, and 0.95 in the case of rice, suggesting little variation in the spatial crop mix of main staple crops over time.

Moreover, the MVDI can be validated against local MV diffusion rates in places where the latter were measured over time, in the spirit of a first-stage test. Historical data on MV use is available for India at the district (admin 2) level from 1960-2000 (ICRISAT, 2013). Table A3 presents estimates of the following regression, showing that the constructed MVDI is well-correlated with actual MV diffusion rates in India:

(2) $${\mathrm{MV}}_{\mathrm{dt}}=\beta {\mathrm{MVDI}}_{\mathrm{dt}}+u_{d}+v_{t}+e_{\mathrm{dt}}$$

where, ${\mathrm{MV}}_{\mathrm{dt}}$ is the area weighted adoption of modern varieties in district d at time t (constructed using district-level data ICRISAT (2013)); ${\mathrm{MVDI}}_{\mathrm{dt}}$ is the constructed MVDI variable in district d at time t; $u_{d}$ are district fixed effects and $v_{t}$ are year fixed effects; and $e_{\mathrm{dt}}$ is the idiosyncratic error term that is clustered at district level. The Indian administrative data on area planted to MVs is only reported for 5 crops, therefore MVDI using EarthStat 2000 areas is only calculated using those crops [i.e. j=5 (rice, wheat, maize, sorghum, millet)]. The definition of MVDI based on EarthStat 1961-1965 areas uses j=3 (rice, wheat, maize).

#### 2.2.2 Alternative MV Definitions

One concern with the definition of MVDI in equation (1) is that the adoption of MVs at the country level ($\mathrm{EGMV}{\mathrm{Area}}_{\mathrm{jct}}$) might be endogenous. To guard against this, consider the following alternative definition which is created using the regional average of MV adoption, leaving out the country where the DHS sampling cluster is located:

(3) $${\mathrm{MVDI}}_{\mathrm{vcrt}}^{-c}=\frac{\sum_{j=1}^{J}\left({\mathrm{CropArea}}_{\mathrm{jvc}}\times {\mathrm{EGMVArea}}_{\mathrm{jcrt}}^{-c}\right)}{\sum_{j=1}^{J}\left({\mathrm{CropArea}}_{\mathrm{jvc}}\right)}$$

where, *v* is a DHS sampling cluster in country *c* in region *r* and *t* is the period of observation. ${\mathrm{EGMVArea}}_{\mathrm{jrt}}$ is the share of area cultivated with crop *j* that is planted with MVs in region *r* at time *t*, and ${\mathrm{CropArea}}_{\mathrm{jvc}}$ is the area cultivated with crop *j* in location *v*, as reported in the global crop maps (which are time invariant) mentioned above. ${\mathrm{EGMV}}_{\mathrm{jcrt}}^{-c}$ refers to the area-weighted regional average for MV adoption for crop j in country c in region r, calculated using all 86 countries in Evenson and Gollin (2003b) after excluding country c. Region r= (Caribbean, Eastern Africa, Middle Africa, Northern Africa, South America, South-Eastern Asia, Southern Africa, Southern Asia and Western Africa). This definition of MVDI based on the leave-out regional average represents a more exogenous proxy for MV diffusion because it is based on MV adoption in neighboring countries.

A second alternative construction of the MVDI imposes additional spatial structure beyond equation (1) by assuming that areas growing more of a crop receive proportionally more MVs per unit area. We first examine this possibility empirically using actual MV diffusion from India (ICRISAT, 2013), where we are able to observe the spatial dynamics of actual MV diffusion for each crop, and confirm this hypothesis for the three crops used to construct the MVDI version that is based on EarthStat 1961-1965. As Figure A4 shows, MV diffusion rates for a crop are positively correlated, across districts, with the relative share of this crop in the local crop mix in 1961-1965. This more spatially structured version of the MVDI (‘Adjusted MDVI’) assumes that MV diffusion for any given crop is highest at locations in which this crop's share in the local crop mix is highest, in a way that preserves the country-level aggregate MV diffusion rate for the crop in each particular year and country. The degree of the skew towards high crop-share areas follows what is observed in the India data in Figure A4. Table A3 shows that the Adjusted MVDI (in Panel B) correlates well with actual MV diffusion rates in India, and has a higher coefficient (closer to the ideal of 1) when compared to the MVDI constructed with equation (1) (Panel A).

### 2.3 Empirical Specification

The main specification in the paper is as follows:

(4) $$y_{\mathrm{ivct}}=\gamma {\mathrm{MVDI}}_{\mathrm{vct}}+u_{v}+Z_{\mathrm{ct}}+X_{\mathrm{ivct}}+e_{\mathrm{ivct}}$$

where, $y_{\mathrm{ivct}}$ is a binary indicator of infant mortality i.e. whether child i born in year t in DHS sampling cluster v in country c died in the first year of life; ${\mathrm{MVDI}}_{\mathrm{vct}}$ is the constructed indicator of MV diffusion in the grid cell to which cluster *v* belongs.^10^ $X_{\mathrm{ivct}}$ is a vector of child-level controls that includes the child’ s sex and a quadratic function of the mother’ s age. The regression controls for DHS cluster fixed effects $u_{v}$, and country-by-year fixed effects $Z_{\mathrm{ct}}$. The DHS sampling cluster fixed effects absorb all time-invariant location characteristics plausibly correlated to both MVDI and health, such as climate, soils, or distance to the capital city. The country-by-year fixed effects flexibly remove national trends, thus controlling for all country-level, time variant variables (economic growth, agricultural policy changes or vaccination campaigns, for example) that might have otherwise biased country-level analyses of the MV-IM relationship.

Our analysis therefore asks whether the change in MV adoption in a given DHS cluster was ahead of or behind the national trend, and whether this deviation in the rate of change was associated with a deviation in the rate of decline of IM risk among children sampled in that DHS cluster. $e_{\mathrm{ivct}}$ refers to the idiosyncratic error term, which is clustered at the admin-1 level (there are 966 state-level administrative zones in the 37 countries in our data) to account for spatial autocorrelation in the treatment variable as well as temporal (serial) correlation in the outcome variable (Bertrand et al., 2004). In addition, our main results in Table 2 include a second set of standard errors that employ two-way clustering at the admin-1 and at the country-year level. This adjusts for spatial autocorrelation that might exist beyond the admin-1 level.

**Table 2:** Impact of modern varieties on infant mortality

	(1)	(2)	(3)
	EarthStat (circa 2000)	SPAM (circa 2000)	EarthStat (1961-1965)
*Panel A: All Children*			
MVDI	−0.0752	−0.0663	−0.0668
	(0.0302)*	(0.0200)**	(0.0208)**
	[0.0325]*	[0.0206]**	[0.0226]**
N	597,247	577,101	581,490
Mean	.1	.1	.1
*Panel B: Females*			
MVDI	−0.0411	−0.0407	−0.0277
	(0.0370)	(0.0277)	(0.0279)
	[0.0391]	[0.0291]	[0.0285]
N	289,183	279,563	281,724
Mean	.096	.095	.097
*Panel C: Males*			
MVDI	−0.0922	−0.0844	−0.1090
	(0.0384)*	(0.0228)**	(0.0244)***
	[0.0398]*	[0.0231]**	[0.0247]***
N	305,379	295,014	297,236
Mean	.11	.11	.11

Note: Each estimate in Table 2 represents γ from the following estimating equation: $y_{\mathrm{ivct}}=\gamma {\mathrm{MVDI}}_{\mathrm{vct}}+u_{v}+Z_{\mathrm{ct}}+X_{\mathrm{ivct}}+e_{\mathrm{ivct}}$ where $y_{\mathrm{ivct}}$ is a binary indicator of infant mortality i.e. whether child i born in year t in DHS sampling cluster v in country c died in the first year of life; $u_{v}$ are cluster fixed effects and $Z_{\mathrm{ct}}$ are country-by-year FE; $X_{\mathrm{ivct}}$ includes quadratic in mother's age (at birth of child) and sex of child; and $e_{\mathrm{ivct}}$ are idiosyncratic errors. Columns report estimates obtained through the use of the three global crop map datasets. The sample is restricted to rural DHS clusters and mothers who report to have never migrated. Standard errors in parentheses are clustered at the sub-national (admin) level, and square brackets are two-way clustered at the admin and country-by-year level. Standard errors in parentheses.

* p<0.10.

** p<0.05 .

*** p<0.001.

The coefficient of interest is γ, which we hypothesize to be negative if faster MV diffusion led to reductions in IM. As the MVDI is a downscaled measure of national MV diffusion data, it offers a prediction of actual MV diffusion at local level. Strictly speaking, we estimate the treatment effects of predicted MV diffusion on infant mortality, but for brevity refer to it as the impact of MVDI or MV diffusion. This should be kept in mind when interpreting the results of the paper.

Since our estimates rely only on subnational deviations from trends (and pooling across countries), this approach offers a significant improvement on cross-country analyses. It dramatically increases sample sizes and data resolution, allowing for more precise statistical estimation and allowing implicit and explicit controls for numerous other potential drivers of IM declines. While our approach does not eliminate all possible causes of potential bias, it greatly reduces the scope for such bias when compared with existing studies on a global scale.

We subject our results to a wide range of robustness tests, which include controlling for indicators of other drivers of IM decline such as maternal education levels or access to public health, controlling for predictors of localized economic growth such as access to trade, removing crop-specific trends that could potentially be driving the association, and limiting the comparison to siblings. A final test guards against the possibility that differences in the rates of MV diffusion across crops in the same country could be influenced by intra-country geographical variation in rates of economic development or health improvements. The test makes use of MVDI values constructed with each country's crop map but with MV diffusion rates taken from and averaged across neighboring countries (excluding the country in question). Since random assignment of MV diffusion across populations is only feasible at local scales, our approach offers the most rigorous quasi-experimental alternative to study this important question on a global scale.

In conducting robustness checks, we estimate a version of equation (4) in which the vector $X_{\mathrm{ivct}}$ includes additional child or DHS cluster-level controls. We also estimate two specifications designed to allow for additional subnational time trends that correlate with geographic features or the local crop mix. Firstly, we estimate:

(5) $$y_{\mathrm{ivct}}=\gamma {\mathrm{MVDI}}_{\mathrm{vct}}+u_{v}+Z_{\mathrm{ct}}+X_{\mathrm{ivct}}+A_{\mathrm{ct}}\times D_{\mathrm{vc}}^{\mathrm{Coast}}+B_{\mathrm{ct}}\times D_{\mathrm{vc}}^{\mathrm{Cities}}+e_{\mathrm{ivct}}$$

where all terms are as in the main regression, with the addition of interactions between country × year fixed effects ($A_{\mathrm{ct}},B_{\mathrm{ct}}$) and the distance of each DHS cluster from the coast $\left(D^{Coast}\right)$ and from cities $\left(D^{Cities}\right)$. This model separates the effect of MV diffusion on IM from any country-specific flexible time trends that differentiate locations on the basis of their distance to coast or urban centers, and therefore flexibly captures much of the local patterns of economic growth within countries. Secondly, we estimate:

(6) $$y_{\mathrm{ivct}}=\gamma {\mathrm{MVDI}}_{\mathrm{vct}}+u_{v}+Z_{\mathrm{ct}}+X_{\mathrm{ivct}}+\sum_{j}\alpha_{j}A_{\mathrm{ct}}^{\left(j\right)}\times \mathrm{Crop}{\mathrm{Area}}_{\mathrm{jvc}}+e_{\mathrm{ivct}}$$

where all terms are as in the main regression, with the addition of interactions between crop specific region year fixed effects $A_{\mathrm{ct}}^{\left(j\right)}$ and the cropped area of each crop j in the location in question, for all crops in the data.

A final consideration is that the restriction of our sample to mothers who have never moved affects the interpretation of results. Our estimate represents the impact on the subsample of children born to mothers in rural areas who never migrated during the MV diffusion. While this sample could potentially be endogenously determined by MV diffusion, we note that our results cannot be driven by changes in sample composition over time. Since each village is surveyed only once, the resulting panel of children comes from a fixed set of mothers who were surveyed. Note that since migrants cannot be linked to village of origin, a similar caveat in interpretation would apply to any study that employs retrospective panels constructed from the DHS surveys.

## 3 Results

### 3.1 Effect on Infant Mortality

Our main results obtained by estimating equation (4) establish that, relative to the national trend, children born when MVs achieved wider diffusion in their clusters were less likely to die in infancy (Table 2, Panel A). The result is robust to using distinct versions of the MVDI derived from the three global crop map datasets (shown across columns 1-3).

Column 1 reports estimates derived by using the EarthStat global crop maps for 11 crops in 2000 (Monfreda et al., 2008). The magnitude of the estimate suggests that an increase of one standard deviation (17 percentage points) in MVDI is associated with a 1.3 percentage point decline in infant mortality (compared to the sample mean of IM of 18% in 1960, and 10% over the 1960-2000 period). Columns 2 and 3 report analogous estimates derived using the SPAM and historical (1961-1965) EarthStat global crop datasets, respectively. The three sets of estimates are similar in both magnitude and precision. For further robustness and heterogeneity analysis, we prefer the MVDI constructed with the crop map in the earliest period (i.e., EarthStat 1961-1965) since the crop shares within a grid cell in later years might be endogenous to subsequent MV adoption for specific crops.

Our empirical strategy does not allow us to directly identify the mechanism through which MVDI decreases IM. The primary candidate mechanisms (assuming an inverse relationship between IM and MV) include an increase in food consumption by mothers in subsistence households, an increase in income by farming households, and a decrease in food prices overall. We explore these mechanisms by way of heterogeneity analysis in Section 3.2.

We further explore whether the effect of MV on IM varies among the world regions represented in our sample. Table 3 compares estimates separately derived in different world regions, using the Earthstat 1961–1965 crop mix specification. Three regions exhibit negative and statistically significant results: Latin America, Africa (whether defined as sub-Saharan Africa or as sub-Saharan African with North Africa), and South Asia (see Table A2 for the full list of countries included in the sample). The magnitude of the effect is larger in Latin America (∼2.5x) and South Asia (∼3x) than in Africa. In Latin America and especially in Africa, the beneficial effect of MVDI on infant health is mostly evident in the case of male infants, while in South Asia the effect appears stronger in female infants. South and Southeast Asia (SSEA) grouped together do not show a significant association of MVDI with a reduction in infant mortality, although the point estimate for the pooled sample is almost identical to the more precisely estimated coefficient for sub-Saharan Africa.

**Table 3:** Impact of modern varieties on infant mortality, by region

	(1)	(2)	(3)	(4)	(5)
	LAC	Africa	SSA only	SSEA	SA only
*Panel A: All Children*					
MVDI	−0.1492	−0.0576	−0.0208	−0.0207	−0.1846
	(0.0590)**	(0.0219)***	(0.0175)	(0.0741)	(0.0929)**
N	76,055	427,907	308,860	77,528	36,487
Mean	.075	.11	.12	.085	.075
*Panel B: Females*					
MVDI	−0.1073	−0.0152	0.0223	−0.1082	−0.2352
	(0.0754)	(0.0296)	(0.0250)	(0.0969)	(0.1283)*
N	36,966	208,198	150,729	36,560	16,625
Mean	.07	.1	.11	.08	.072
*Panel C: Males*					
MVDI	−0.2155	−0.1006	−0.0637	0.0630	−0.1314
	(0.0831)**	(0.0252)***	(0.0254)**	(0.1052)	(0.1199)
N	38,680	219,055	157,535	39,501	18,519
Mean	.081	.12	.12	.092	.08

Note: Each estimate in Table 3 represents γ from the following estimating equation run for each region separately: $y_{\mathrm{ivct}}=\gamma {\mathrm{MVDI}}_{\mathrm{vct}}+u_{v}+Z_{\mathrm{ct}}+X_{\mathrm{ivct}}+e_{\mathrm{ivct}}$ where $y_{\mathrm{ivct}}$ is a binary indicator of infant mortality i.e. whether child i born in year t in DHS sampling cluster v in country c died in the first year of life; $u_{v}$ are cluster fixed effects and $Z_{\mathrm{ct}}$ are country-by-year FE; $X_{\mathrm{ivct}}$ includes quadratic in mother’ s age (at birth of child) and sex of child; and $e_{\mathrm{ivct}}$ are idiosyncratic errors clustered at subnational (admin) level. Columns report estimates obtained through the use of the three global crop maps discussed in the main text. The sample is restricted to rural DHS clusters and mothers who report to have never migrated. Latin America and the Caribbean (LAC) includes 5 countries (Bolivia, Colombia, Dominican Republic, Haiti and Peru), Africa includes 27 countries (Egypt, Morocco and SSA countries), sub-Saharan Africa (SSA) includes 25 countries (Benin, Burkina Faso, Central African Republic, Camerron, Congo DR, Cote d’Ivoire, Ethiopia, Ghana, Guinea, Kenya, Liberia, Malawi, Mali, Namibia, Niger, Nigeria, Rwanda, Senegal, Sierra Leone, Swaziland, Tanzania, Togo, Uganda, Zambia and Zimbabwe), South and South East Asia (SSEA) includes 5 countries (Cambodia, Philippines and SA countries) and South Asia (SA) includes 3 countries (Bangladesh, India and Nepal). Standard errors in parentheses.

* p<0.10.

** p<0.05.

*** p<0.01.

#### 3.1.1 Results by Child Sex

Across all three crop datasets, the estimated impact on IM among female infants is smaller in magnitude than the pooled effect across sexes and is statistically insignificant (Table 2 panel B). Mortality among male infants, on the other hand, displays a larger and highly significant association (Table 2 panel C). Coefficient estimates imply that males born when MVDI in their cluster is one standard deviation higher than the national trend benefit from a 1.4–1.9 percentage point reduction in IM risk (as compared to an average IM of 11% across the entire sample of males). These results suggest that if MVDI does in fact improve infant health, whether through increased caloric intake or higher incomes, the effect is greater among male than female infants.

To test whether the sex-differentiated salubrious effects of MVs occur in utero, we estimate the impacts of MVDI on the infant male-to-female sex ratio of the children in the sample (i.e. live births), but find only weak evidence of an increase in male births (Table A4).

We explore heterogeneity by child sex by undertaking two approaches. First, we examine the pattern in the marginal effect of MVDI across child sex according to each country's Gender Parity Index (World Bank, 2015). The GPI is calculated as the ratio of girls to boys enrolled at primary and secondary levels in public and private schools, such that values closer to unity represent more parity. We take the average GPI from 1970-2000 for the countries in our sample and classify the countries as high or low gender parity depending on whether they fall above or below the median (the median value of GPI is 0.74). After dividing the sample according to the median GPI threshold, we test whether the marginal effect of MVDI exhibits the same patterns by child sex across low-parity and high-parity countries. Table 4 shows the results, weighting observations so that coefficients represent the marginal effect in the average country. Two aspects of these results are particularly relevant. First, column (1) shows that the main effect of MVDI on infant mortality is present in both low- and high-GPI groups. Column (2) shows that a salubrious effect of MVDI on infant mortality risk among girls is evident only in countries with relatively more gender parity. This supports the idea that a discriminatory mechanism partly explains the different impact of MVDI across boys and girls. Column (3) makes it clear that the benefit of MVs on boys is consistent across countries, regardless of their GPI.

**Table 4:** Heterogeneous effects of MVDI on IM, by gender parity index

	(1)	(2)	(3)
	All	Girls	Boys
*Panel A: All Regions*			
MVDI	−0.0583	−0.0103	−0.0896
	(0.0208)***	(0.0262)	(0.0284)***
MVDI × above median GPI	−0.0595	−0.1368	−0.0268
	(0.0648)	(0.0707)*	(0.0807)
N	563,820	273,054	288,240
Mean	.097	.092	.1
*Panel B: sub-Saharan Africa only*			
MVDI	−0.0566	−0.0112	−0.0830
	(0.0223)**	(0.0278)	(0.0313)***
MVDI × above median GPI	−0.1114	−0.0976	−0.0970
	(0.0654)*	(0.0758)	(0.1044)
N	308,860	150,729	157,535
Mean	.12	.11	.12

Note: Each estimate in Table 4 represents γ and θ from the following estimating equation: $y_{\mathrm{ivct}}=\gamma {\mathrm{MVDI}}_{\mathrm{vct}}+\theta {\mathrm{MVDI}}_{\mathrm{vct}}\times W_{\mathrm{vc}}+u_{v}+Z_{\mathrm{ct}}+X_{\mathrm{ivct}}+e_{\mathrm{ivct}}$ where $y_{\mathrm{ivct}}$ is a binary indicator of infant mortality i.e. whether child i born in year t in DHS sampling cluster v in country c died in the first year of life; ${\mathrm{GPI}}_{c}$ is equal to one if country c has above median average Gender Parity Index from 1970-2000 (calculated from World Bank (2015)); $u_{v}$ are cluster fixed effects and $Z_{\mathrm{ct}}$ are country-by-year fixed effects; $X_{\mathrm{ivct}}$ includes quadratic in mother’ s age (at birth of child) and sex of child; and $e_{\mathrm{ivct}}$ are idiosyncratic errors clustered at subnational (admin) level. MVDI is calculated using the EarthStat 1961-1965 crop map data. Standard errors in parentheses.

* p<0.10.

** p<0.05.

*** p<0.01.

Given that GPI is correlated to region (Latin America is high-GPI and South Asia is low-GPI) we look for further evidence of a GPI gradient in the marginal effect of MVDI by looking only within one region. The only region in the sample with enough countries to measure the marginal effect of MVDI across low-GPI and high-GPI groups is sub-Saharan Africa. We divide the countries in the region by the median 1970-2000 average GPI, and produce the results in Panel B of Table 4. Within sub-Saharan Africa, the pattern of MVDI marginal effects remains. All countries demonstrate strong marginal effects of MVDI regardless of GPI in the case of boys and the pooled sample. The sign and magnitude of the coefficients on girls suggests that MVDI may have an effect on girls only in high GPI countries, but it is not statistically significant. While these results suggest that gender discrimination may have a role in explaining the different effects between boys and girls, the statistical pattern is not strong.

We conclude that while there is some evidence that the marginal effect of MVDI on girls follows a gradient consistent with gender discrimination, the evidence is not statistically definitive. Meanwhile, the effect on boys is remarkably consistent across these tests and across variation in gender parity, giving support to the underlying biological difference between males and females as the mechanism.

#### 3.1.2 Within-Parity and Within-Mother Estimates

We use two approaches to further bolster confidence in the causal identification of an effect of MV on IM in our model. First, column 1 in Table A5 estimates a model that includes a flexible control for the birth order of each child (fixed effects for birth order). This test is motivated by evidence of linkages between resource allocation to children and their birth order in some developing country contexts.^11^ The resulting estimates are almost unchanged from those in the main analysis in Table 2, showing no evidence of preference by birth order.

Secondly, c column 2 reports estimates from a highly demanding model that includes mother fixed effects. Including these fixed effects is equivalent to comparing only children born to the same mother, thus separating the impacts of MVDI from all observable and unobservable maternal characteristics that are time-invariant. Remarkably, the basic results of the model remain qualitatively unchanged in this highly stringent specification. The coefficients on pooled genders and on males remain negative, although they are smaller in magnitude compared to the main results, and imprecisely estimated. In the case of males the coefficient remains statistically significant.

### 3.2 Potential Mechanisms

#### 3.2.1 Heterogeneity by Socioeconomic Characteristics

Where the goal is to inform policy, it is highly relevant to understand whether the benefits of MVDI tend to accrue to poorer households as well as wealthier ones. To elucidate this issue, we estimate regressions that examine differences in the impact of MVDI on IM on households that differ in whether the mother engages in agricultural wage labor, whether the mother is illiterate, and a poverty score. Table 5 reports estimates of these regressions for all children (top panel), females (middle panel) and males (bottom panel). Each column in each panel reports the results of a separate regression that uses the poverty characteristic reported at the top of the column, and reports the point estimates of MVDI (corresponding to the coefficient γ, the characteristic in question δ, and their interaction θ).

**Table 5:** Heterogeneous impacts of modern varieties on infant mortality, by occupation, education and income

	(1)	(2)	(3)
	Occupation (Ag. wage worker)	Education (Not literate)	Wealth (Poverty score)
*Panel A: All Children*			
MVDI	−0.0695	−0.0401	−0.0702
	(0.0239)***	(0.0203)**	(0.0210)***
Characteristic	0.0113	0.0131	0.0053
	(0.0042)***	(0.0020)***	(0.0013)***
MVDI ×	−0.0295	−0.0185	−0.0056
Characteristic	(0.0109)***	(0.0056)***	(0.0034)*
N	546,618	441,077	562,129
*Panel B: Females*			
MVDI	−0.0289	0.0023	−0.0346
	(0.0313)	(0.0265)	(0.0283)
Characteristic	0.0090	0.0128	0.0059
	(0.0047)*	(0.0025)***	(0.0016)***
MVDI ×	−0.0221	−0.0154	−0.0097
Characteristic	(0.0157)	(0.0076)**	(0.0044)**
N	265,720	213,285	272,210
*Panel C: Males*			
MVDI	−0.1097	−0.0850	−0.1094
	(0.0274)***	(0.0271)***	(0.0254)***
Characteristic	0.0135	0.0135	0.0051
	(0.0056)**	(0.0029)***	(0.0018)***
MVDI ×	−0.0403	−0.0224	−0.0035
Characteristic	(0.0178)**	(0.0083)***	(0.0049)
N	279,259	225,396	287,409

Note: Each estimate in Table 5 represents γ, δ and θ from the following estimating equation run for each mother's characteristic (occupation, education and income) separately: $y_{\mathrm{imvct}}=\gamma {\mathrm{MVDI}}_{\mathrm{vct}}+\delta W_{\mathrm{imvct}}+\theta {\mathrm{MVDI}}_{\mathrm{vct}}\times W_{\mathrm{imvct}}+u_{v}+Z_{\mathrm{ct}}+X_{\mathrm{imvct}}+e_{\mathrm{ivct}}$ where $y_{\mathrm{ivct}}$ is a binary indicator of infant mortality i.e. whether child i born in year t in DHS sampling cluster v in country c died in the first year of life; $W_{\mathrm{ivct}}$ is a characteristic of the mother interacted with the MVDI; $u_{v}$ are cluster fixed effects and $Z_{\mathrm{ct}}$ are country-by-year fixed effects; $X_{\mathrm{ivct}}$ includes quadratic in mother's age (at birth of child) and sex of child; and $e_{\mathrm{ivct}}$ are idiosyncratic errors clustered at subnational (admin) level. MVDI is calculated using the EarthStat 1961-1965 crop map data. $W_{\mathrm{imvct}}$ in column 1 is coded as 1 if the mother is an agricultural wage worker, 0 otherwise. In column 2, it is coded as 1 if the mother is not literate, 0 otherwise. In column 3, $W_{\mathrm{imvct}}$ represents a degree of poverty computed by an index that includes socio-economic household traits like education, marriage status, type of floor, roof and wall, owernship of agricultural assets such as livestock, land and animal cart. The score is created only for rural areas and the scale is reversed so that higher values indicate fewer assets. Standard errors in parentheses.

* p<0.10.

** p<0.05.

*** p<0.01.

As before, the coefficients of MVDI on all children and on males are all negative. In addition, and predictably, the coefficients on the poverty characteristics are all positive and statistically significant, indicating that infants born to mothers with the characteristics considered here are at higher risk of mortality. Most importantly for this discussion, the coefficients on the interaction terms are also all negative and almost all statistically significant, indicating that increases in MVDI are likely to lead to larger declines in IM in poorer households. For example, the results reported in column 1, top panel, indicate that while having 10% more crops planted to MVs reduces IM by 0.7 percentage points for children with mothers who are not engaged in agricultural wage labor, the decline is larger by 0.3 percentage points for mothers who are (a total effect of 0.695+0.295=0.99 percentage points).

#### 3.2.2 Heterogeneity by Distance to Cities

As discussed in the main text, our identification strategy only measures impacts of MVDI that occur due to increases in farmers’ consumption and incomes or because of localized reductions in food prices. As food production increases with the diffusion of MVs, food prices are likely to decline less in places with access to large markets, and decline more in places where transport costs to markets are high (if the food must be sold in local markets, the price effects of large production increases are likely stronger). While these price decreases may reduce incomes for some farmers, they lead to improved welfare for the rest of the population. Our setup does not allow us to formally test whether the price mechanism is an important driver of the mortality reduction. However, we examine heterogeneity in MVDI impacts related to the degree of market connectivity by way of illustrating the potential role of price effects. We test this by comparing the impacts of MVDI in areas near to urban centers to those that are farther away.

Table A6 reports estimates of a regression that includes the interaction of MVDI with a control for distance to urban centers. Column 1 uses the distance to urban centers with population exceeding 500,000 people, while column 2 uses distance to urban centers exceeding one million people. The interaction terms are negative (for the full sample and for males), indicating that effects of MVDI on IM are stronger farther away from cities, where markets are more likely to be disconnected and price effects are stronger. In the case of male children, for which the interaction term is more precisely estimated, every 100 km increase in distance increases the effect size of MVDI by around one-fifth. While these results cannot establish that a food price mechanism is a partial driver of mortality declines, they are consistent with such an interpretation. It is important to clarify that distance to cities is likely to be correlated with a range of other characteristics that may influence the impact of MVs on IM, so these results should be interpreted with some care and should only be viewed as an illustration of heterogeneous effects.

#### 3.2.3 Effect on Malnutrition

As a test of whether the diffusion of modern varieties reduced infant mortality by improving child nutrition, Table 6 reports estimates of the association between the MVDI and four measures of undernutrition: severe stunting (defined as being more than three standard deviations below the age-specific mean of height-for-age), severe wasting (three standard deviations below median weight-for-height), severe underweight (three standard deviations below median weight-for-age), and low birthweight (less than 2,500 grams). The sample of children is smaller than the main sample because anthropometric measures are only measured for children that are younger than five years at the time of the survey.

**Table 6:** Impact of modern varieties on malnutrition

	(1)	(2)	(3)
	EarthStat (circa 2000)	SPAM (circa 2000)	EarthStat (1961-1965)
*Panel A: Severe stunting*			
MVDI	−0.6708	−0.1923	−0.6124
	(0.2608)**	(0.1774)	(0.3022)**
N	51,520	50,257	50,573
Mean	.18	.18	.18
*Panel B: Severe wasting*			
MVDI	−0.0440	−0.0864	−0.0401
	(0.0724)	(0.0517)*	(0.0797)
N	53,182	51,809	52,177
Mean	.018	.018	.018
*Panel C: Severely underweight*			
MVDI	−0.2334	−0.1125	−0.0478
	(0.1744)	(0.1296)	(0.1512)
N	51,520	50,257	50,573
Mean	.088	.086	.087
*Panel D: Low birthweight*			
MVDI	−0.0312	−0.0913	−0.5816
	(0.5509)	(0.3515)	(0.9205)
N	8,779	8,623	8,634
Mean	.12	.12	.12

Note: Each estimate in Table 6 refers to γ from the following estimating equation: $y_{\mathrm{ivct}}=\gamma {\mathrm{MVDI}}_{\mathrm{vct}}+u_{v}+Z_{\mathrm{ct}}+X_{\mathrm{ivct}}+e_{\mathrm{ivct}}$ where $y_{\mathrm{ivct}}$ is a binary indicator of malnutrition i.e. child *i* born in year *t* in DHS cluster *v* in country *c* had a height-for-age Z score less than 3 standard deviation below median (severe stunting); weight-for-height Z score less than 3 standard deviation below median (severe wasting); weight-for-age Z score less than 3 standard deviation below median (severely underweight); or birthweight was less than 2,500 grams (low birthweight); $u_{v}$ are cluster fixed effects and $Z_{\mathrm{ct}}$ are country-by-year fixed effects; $X_{\mathrm{ivct}}$ includes quadratic in mother's age (at birth of child), sex of child and a dummy for the child's age; and $e_{\mathrm{ivct}}$ are idiosyncratic errors clustered at subnational (admin) level. Columns report estimates obtained through the use of the three global crop maps. The sample is restricted to rural DHS clusters and mothers who report to have never migrated. Standard errors in parentheses. ***p<0.01

* p<0.10.

** p<0.05.

The results suggest a negative association between MVDI and malnutrition indicators in eleven of twelve specifications. We note that these estimates are underpowered, since they are limited to one decade of data (DHS surveys began in the early 1990s, and this analysis ends in 2000 with the MV data). In the case of severe stunting, results are statistically significant for two of the three crop maps, and magnitudes indicate that as a location's MVs increase by 10 percentage points, children's risk of being severely stunted in that area decreases by 6–7 percentage points (using the EarthStat 2000 or 1961-1965 crop maps in columns 1 and 3). The coefficient on the MVDI constructed with the SPAM dataset (column 2) is also negative, but is smaller in magnitude and not statistically significant. Results for the other malnutrition indicators are consistently negative, but not statistically significant.

### 3.3 Robustness Tests and Additional Analysis

We conduct a variety of robustness tests in order to further scrutinize the causal interpretation of the results. First, we ascertain that results are not sensitive to different sampling weight choices. Secondly, we account for the expansion of public health services that was underway during the study period. Thirdly, we consider the possibility that unobserved localized trends of both agricultural and non-agricultural economic growth could be driving the correlation between MV and IM. Fourthly, we show robustness to working with two alternative versions of the MVDI. Next, we test whether effects of MVDI are evident when looking only at years around the arrival of MVs. Further, we examine sensitivity to including migrants in the estimating sample. Finally, we conduct two placebo tests by both checking effects in urban areas and subjecting the model to randomization tests. Figure A6 summarizes the various estimates obtained from robustness checks.

#### 3.3.1 Sampling and Population Weights

The main results presented in Table 2 represent the average treatment effect of MVDI among the children in our sample. However, if the impact of MV on IM varies in ways that correlate with sampling design, our results would not be representative for all children in our sample countries if we do not explicitly take into account the sampling procedure within each country, and adjust for population across countries. This consideration leads some scholars to provide weighted estimates when using multi-country and multi-survey DHS data (Vollmer et al., 2014, Burke et al., 2015, Burke et al., 2016, Vogl, 2016, Heft-Neal et al., 2018). However, the use of weights does not resolve concerns over heterogeneous treatment effects, nor is the choice of weights obvious. DHS sampling weights are only designed to achieve representativeness within a particular country and survey round, rather than across countries or across surveys, leading some scholars to prefer reporting unweighted estimates (Baird et al., 2011, von der Goltz and Barnwal, 2019). Moreover, our exercise uses a subsample (rural mothers who report to have never moved), which further complicates the question on how to use the weights to draw population-level inference.

Nevertheless, we test the sensitivity of our results to including weights defined based on the sample principles applied in similar papers that use multi-country DHS data. Table A7 presents the same specification as Table 2, but now weighing observations by the product of the DHS sampling weights and a population adjustment factor (equal to the country's rural population, $N_{c}^{r}$, divided by the country sample size in our regressions of mothers in rural areas who have never moved, $n_{c}^{r}$). Note that the denominator in the weight is the sample size across all the DHS surveys for that country, $\sum_{s}n_{c}$, which adjusts the weight for the fact that countries have had different numbers of DHS surveys. Since we are using the rural subsample of the DHS in each country, we re-center the DHS sampling weights in each country-survey year to return them to mean one, $h^{\prime }$, as in DHS survey weights. Thus the weights used are $w=h^{\prime }\times N_{c}^{r}/\sum_{s}n_{c}^{r}$.

The results obtained from weighted regressions shown in Table A7 are qualitatively very similar to the unweighted results in Table 2. The estimates in columns 1 and 3, from EarthStat 2000 and EarthStat 1961-1965, are similar in magnitude and always larger than those in Table 2, as well as statistically significant. The coefficient estimates in column 2, using the SPAM 2000 data, retain a negative sign, but are smaller in magnitude than their analogues in Table 2, and are not statistically significant. Nevertheless, the similarity between the weighted and unweighted results for two of the crop maps suggest that our main results are robust to using sample weights. Given the methodological uncertainty in the literature over the appropriate use of weights in multi-country DHS data, we opt for presenting the unweighted results as the main result in the paper.

#### 3.3.2 Expansion of Public Health and Maternal Education

Improvements in maternal education as well as increases in access to public health services (maternal, neonatal and child health interventions in particular) are leading determinants of infant mortality reductions (Cutler et al., 2006). This study of the effect of MVDI on infant mortality does not in any way contradict the importance of these factors. However, since access to public health services and to education increased over the period of our analysis, it is important to note that a correlation between the sub-national diffusion of MVs and public health or education could potentially bias our estimates. We therefore explore whether there is reason to suspect bias.

While the DHS records public health indicators, they are only available at the time of survey, so that we cannot correctly assign to each child the health services accessible by the mother when the child was born. Given this constraint, we use DHS data to construct cluster-level indicators of access to maternal health services at time of survey, and directly test for correlation of these indicators with MVDI at the cluster level. Estimates of regressions are reported in Table A8 for each of the three global crop maps used in the analysis. There is no indication of a systematic association between the health measures in question and MVDI. Antenatal care is the only measure that shows a positive association with MVDI in two out of three crop maps. Other coefficients reflect mostly insignificant associations that vary in sign across crop maps and public health measure. Note that even an association between MVDI and a measure of public health would not invalidate our approach, since improved health behaviour could itself be an outcome of increased crop productivity and income. In this sense, the test is too strong.

#### 3.3.3 Accounting for Local Patterns of Economic Growth

Another potential threat to causal interpretation of our results lies with the possibility that sub-national improvements in both MV diffusion and IM could both be driven by localized variation in the rate of economic development that does not arise from the diffusion of MVs. If for such extraneous reasons, incomes increased at higher rates in certain subnational regions, one might be concerned that they lead to both declines in IM as well as higher ability to invest in improved seeds and associated inputs. This could lead us to incorrectly infer a causal connection between the two variables.

Local incomes are not observed during our study period at the required spatial and temporal resolution, making it difficult to fully account for this possibility. However, we subject our model to several robustness tests. The first test adds sub-national administrative region-by-year fixed effects, which tests whether changes in MVDI rates across clusters in the same sub-national region are correlated with rates of change in IM, relative to the sub-national flexible trend in mortality. The result is reported in column 1 of Table A9, and shows that point estimates are similar to those estimated in the baseline specification.

The second test is defined in equation (5), in which we control for interactions between country-specific flexible time trends and geographical attributes of each location that are often predictive of economic growth, namely distance to the coast and distance to cities. The results, reported in column 2 of Table A9, are nearly identical in size and significance to those obtained in Table 2.

A similar potential concern is that the local crop mix itself could have an impact on declines in IM that is not due to the diffusion of MVs, but some other attribute of the crop mix that leads to increased agricultural development or income growth. For example, one might imagine that differing trends in the global prices of specific crops create different trends in incomes for some locations. If such price trends were correlated with MV diffusion rates across crops, the observed effect on IM might be due to price changes rather than by MV diffusion. Column 3 adds interactions between each crop's area share and crop-specific year fixed effects, as well as interactions between each crop's area share and crop-specific country fixed effects. The broad pattern of the result remains unchanged. We also test for robustness of our estimates to the inclusion of interactions between flexible country-specific time trends and the relative areas of each crop, as shown in equation (6). The results, reported in column 4 of Table A9, are again very similar to those obtained from the basic model in Table 2.

#### 3.3.4 Alternative MVDI

We present results from two alternative constructions of the MDVI, described in more detail above in section 2.2.2. Table 7 reports estimates of the effect of MVDI on IM using a version of the MVDI constructed using the regional averages of each crop's MV diffusion, while excluding the country itself. We run three versions of the “leave one out” test in which the alternative MVDI is constructed using either (1) all neighbouring countries; (2) all countries in the region; or (3) all countries in the global sample (always leaving the country in question out of the average). This provides a more exogenous proxy of MV diffusion since it does not allow country-specific factors affecting both MV diffusion and health improvements to enter the MVDI construction. The estimates in columns 2-4 are from a stringent specification that also controls for regional trends in crop area, in an analogous way as was discussed in Table A9. The results show that the impact of MVDI remains similar in size and as statistically significant as in the original benchmark estimation in Table 2 (replicated in Table 7, column 1). Results are very stable across the three different ways of choosing the group of countries used to construct the alternative MVDI. We present the more conservative results in Table 2 as our preferred estimates.

**Table 7:** Impact of modern varieties on infant mortality using alternative MVDI constructed with different EGMV averages, controlling for regional geographic trends

	(1)	(2)	(3)	(4)
	Country EGMV	Subregional EGMV	Regional EGMV	Global EGMV
*Panel A: All Children*				
MVDI	−0.0668	−0.1093	−0.1011	−0.2032
	(0.0208)***	(0.0418)***	(0.0632)	(0.0958)**
N	581,490	394,564	394,564	394,564
Mean	.1	.1	.1	.1
*Panel B: Girls*				
MVDI	−0.0277	−0.0626	−0.0537	−0.1211
	(0.0279)	(0.0491)	(0.0754)	(0.1035)
N	281,724	191,297	191,297	191,297
Mean	.097	.097	.097	.097
*Panel C: Boys*				
MVDI	−0.1090	−0.1346	−0.1251	−0.2078
	(0.0244)***	(0.0490)***	(0.0764)	(0.1174)*
N	297,236	201,740	201,740	201,740
Mean	.11	.11	.11	.11

Note: Estimates in Table 7 column 1 represents γ from the following estimating equation: $y_{\mathrm{ivct}}=\gamma {\mathrm{MVDI}}_{\mathrm{vct}}+u_{v}+Z_{\mathrm{ct}}+X_{\mathrm{ivct}}+e_{\mathrm{ivct}}$ and those in columns 2-4 represents γ from the following estimation equation: $y_{\mathrm{ivct}}=\gamma {\mathrm{MVDI}}_{\mathrm{vct}}^{\prime }+u_{v}+Z_{\mathrm{ct}}+X_{\mathrm{ivct}}+\sum_{j}\alpha_{j}A_{\mathrm{rt}}^{\left(j\right)}\times \mathrm{Crop}{\mathrm{Area}}_{\mathrm{jvc}}+e_{\mathrm{ivct}}$ where $y_{\mathrm{ivct}}$ is a binary indicator of infant mortality i.e. whether child i born in year t in DHS sampling cluster v in country c died in the first year of life; $u_{v}$ are cluster fixed effects and $Z_{\mathrm{ct}}$ are country-by-year FE; $\sum_{j}\alpha_{j}A_{\mathrm{rt}}^{\left(j\right)}$ refers to interactions between crop-specific region year fixed effects $A_{\mathrm{rt}}^{\left(j\right)}$ and the cropped area of each crop j in the location in question, for the three crops in the EarthStat 1961-1965 crop map data (maize, rice and wheat); $X_{\mathrm{ivct}}$ includes quadratic in mother's age (at birth of child) and sex of child; and $e_{\mathrm{ivct}}$ are idiosyncratic errors clustered at sub-national (admin) level. MVDI is calculated using the EarthStat 1961-1965 crop map data, using EGMV from the country the child was born in. Column 1 reports the estimate from the baseline specification from Table 2. ${\mathrm{MVDI}}^{\prime }$ refers to the adjusted MVDI that is calculated using subregional EGMV averages excluding the observation's country (column 2); regional EGMV averages calculated excluding the observation's country (column 3); and global EGMV from 86 countries (column 4). The sample is restricted to rural DHS clusters and mothers who report to have never migrated. Standard errors in parentheses.

* p<0.10.

** p<0.05.

*** p<0.01.

As discussed earlier, an implicit assumption in the construction of MVDI is that that all parts of a country growing a particular crop receive the same dose of crop-specific national MV. Table A10 reports estimates of the effect of MVDI on IM using an Adjusted MDVI which spreads MVs subnationally for a given crop towards areas where that crop is a larger share of area planted than other crops in the analysis. The skew of MVs towards high crop share areas is set to follow the pattern observed in India in Figure A4. Column 1 reports the results in Table 2 obtained with the original MVDI built with EarthStat 1961-1965 data, while column 2 reports results obtained with the adjusted MVDI (also built with EarthStat 1961-1965 crop areas). The results show that the effects of MVDI on infant mortality are very similar in magnitude and significance across the two versions of MVDI.

#### 3.3.5 Years around MV arrival

In order to test the effect of MV arrival using cohorts of children as similar to each other as possible, we restrict the sample to 10 and 15 years within first adoption in panels A and B, respectively, and define the “moment of MV adoption” at the cluster level in three different ways: MV > 0% in column 1, >5% in column 2 and >10% in column 3. We focus on the subsample of boys given that the estimated MVDI impact is strongest on them.

The estimates are shown in Table A11. Estimates in Panel A are smaller and not always statistically significant, but we note that the sample is reduced in size by 55-65% (compared to N = 297,326 for boys using EarthStat 1961-1965 in Table 2). Estimates in Panel B (with a larger sample size) are more similar to our full sample estimates, and statistically significant in Columns 1-3. The point estimate when limiting the data to 15 years of arrival and a threshold of 10% of MVs is identical to the analogous coefficient in the main result, despite employing only about 60% of the data. Overall, these specifications limiting the sample to the years around first arrival of MVs in a DHS cluster show very similar patterns compared to using all of the data.

#### 3.3.6 Including Migrants

The main regressions are estimated using the sample of women who have never migrated, in order to ensure the historical MVDI in their location accurately reflects the circumstances experienced during early life stages of their children. As a robustness test, we also estimate our model on the full DHS sample. The results, reported in Table A12, are smaller in magnitude, but retain the same pattern (and statistical significance in the case of Earthstat 1961-1965 data) of those obtained with the sample of non-migrants. Note that the smaller magnitude of the coefficients is consistent with the measurement error that may result from incorrectly assigning the MVDI in the current cluster of residence to the MVDI exposure of children born before the migration occurred.

#### 3.3.7 Urban Areas

All estimates reported thus far were obtained by using the rural sample of the DHS. While the DHS does not directly report whether a household is engaged in farming, it does distinguish between rural and urban settlements. Urban households are less likely to be engaged in farming, making it less likely that their incomes will rise as a result of the diffusion of MVs in their areas. In addition, since markets near urban centers are likely to be well connected, the diffusion of MV in the local area is also unlikely to reduce food prices in relation to other urban areas. Finally, urban areas are likely to have far less cropland, such that a percentage change in MVDI is likely to have a small effect on overall food availability for the urban population. It is therefore less likely that MVDI will affect IM for urban households in ways that will be captured by our analysis. Estimating the association in the urban sample therefore provides a kind of “placebo test” for the overall model and estimation approach. Estimates obtained for the rural and urban sample are reported side by side in Table A13 and confirm this prediction: no significant association is detected between MV and IM in the urban sample, and the point estimates are significantly smaller.

#### 3.3.8 Randomization Tests

As a further test against the possibility that our results are driven by spurious associations or the structure of the data, we conduct two randomization tests. The first re-estimates the main model after random assignment of MDVI values across clusters within a country. The second randomly re-assigns the MV variable across crops within a country in order to construct a placebo MVDI that replaces the MVDI variable in equation (4). Figure A5 plots the distribution of the coefficient estimates from 10,000 reshufflings, in comparison to the estimate obtained by using the actual MVDI values. The vertical line indicates that actual estimate of γ obtained by using the EarthStat 1961-1965 cropped area dataset (reported in Table 2). The distribution of γ is centered close to zero, as would be expected, and indicates that the likelihood that our point estimate could have resulted by chance is unlikely (p<0.001 for the randomization across clusters, and p<0.05 for the randomization of MV data across crops).

#### 3.3.9 Recall Bias

The DHS interviews mothers up to the age of 49, meaning that some mothers may be required to recall births that took place decades before the time of the survey. The timing and survival status of past births are highly salient events in a mother's life, suggesting that recall errors are less likely to be a concern than for other types of recalled data. This is especially true since our research design only relies on accurately placing the year of birth within 5-year intervals. Moreover, errors in recalling the survival status of past births could only potentially bias our estimates if it is systematically correlated with variation in IM or MV trends within countries. Nevertheless, it is worth considering whether errors in recalling the timing or survival of distant past births could bias our findings.

We conduct several direct tests of the possibility that recall error could be affecting our estimates. First, we re-estimate our regression on sub-samples in which recall bias is less likely to occur and examine whether the estimates are substantially affected. We report the resulting estimates in Table A14. In panel A, we limit the sample to births that occurred in the latter half of our study period, i.e. post 1980. In panel B, we limit the sample to mothers who were below 40 years of age when they were surveyed (Khatun et al., 2018, Espeut and Becker, 2015, Beall and Leslie, 2014). In panel C, we limit the sample to educated mothers, who are less likely to commit recall error (Beall and Leslie, 2014). In panel D, we limit the sample to births occurring less than 20 years before they are reported in the survey. All four resulting sets of estimates are similar in statistical significance, direction and magnitude to our benchmark results obtained with the full sample. This similarity provides strong evidence against the possibility that recall bias may be affecting our results. In panels E and F we perform two additional tests in which we directly control for the recall year or the recall period, respectively, in the regression (Khatun et al., 2018). In panel G we weigh observations inversely to their length of recall. Once again, the estimates remain very similar to our benchmark results, further strengthening our confidence that recall error is not affecting our results.

#### 3.3.10 Pre-trends and other Threats to Identification

Recent work on Bartik instruments has identified two threats that are relevant in the case of MVDI (Goldsmith-Pinkham et al., 2020). First, the initial cropping pattern may be correlated with other outcome variables, such as age and education of the mother, which may in turn affect infant mortality. We address this concern by controlling for mother's age in our regressions (Table 2) and by running regressions that only compare siblings and thus eliminate any effect of time-invariant characteristics of the mother (Table A5).

A second threat relates to concerns about different pre-trends in infant mortality. Table A15 tests whether trends in infant mortality differ between DHS clusters that will eventually experience an increase in MVs to those that will not, even before MVs begin to diffuse. The test involves regressing the residuals from the main specification in Table 2 on future MVDI (next time period) (). The estimated coefficient is statistically insignificant, meaning that there is no evidence of differences in pre-trends in the IM variable in areas that will adopt more MVs in the future compared to areas that do not.

## 4 Conclusion

In the year 2000, around 114 million children were born per year in the developing world (United Nations Population Division, 2015), while the population-weighted average of crops planted to MVs was 63%. If our estimated effects of predicted local MV diffusion apply to the entire population, they would suggest that this level of MV diffusion and associated Green Revolution technologies reduced the infant mortality rate by 2.4–5.3 percentage points, which translates into around 3–6 million infant deaths averted per year by the year 2000. An important caveat is that our estimates are derived from the sample of rural families who have never migrated, and we cannot directly test whether these generalize to all families.

If the average MV diffusion rate in SSA went up from around 30% in 2010 (Walker and Alwang, 2015) to South Asian levels (around 60%), our estimates using MVDI (EarthStat 1961-65) imply that IM would decline by 6.24 per 1,000 live births if the treatment effects of broader adoption in SSA followed the effect observed so far from limited adoption in SSA (Table 3, Column 3), and by 20.04 if the effects were more akin to the global average (Table 2, Column 3). The former estimate can be interpreted as providing a lower bound, for instance, assuming persistence in the lack of complementary inputs in SSA, when compared to the global average.

At the global level, our estimates imply that an increase in MV adoption from 0 to 50% leads to a decline in IM by 33-38 deaths per 1,000 children. For comparison, Bharadwaj et al. (2020) estimate it to be 15 deaths per 1,000 children using data from India, and Gollin et al. (2018) find it to be about 45 deaths using data from 87 countries. The fact that our estimate is between the magnitudes of two other papers that use different methodologies and a different sample of countries strengthens the claim of a generalized result between MV diffusion and infant mortality.

Three comments on the interpretation of these results are in order. First, since our estimates are based on differences in the rates of IM declines across DHS clusters in the same country, they can only capture those impacts of MV diffusion that are localized in nature. For example, the impacts of uniform declines in food prices across an entire country would be “missed”by our analysis. Only localized relative changes in income and food prices would be captured, meaning our analysis may under-estimate the true impact. We note, however, that imperfect market linkages in developing countries make spatially localized effects on prices quite likely (Van Campenhout, 2007, Gonzalez-Rivera and Helfand, 2001, Ravallion, 1986).

Second, our indicator tracks replacement of traditional crop varieties with modern varieties. Additional crop yield and human welfare benefits would be expected as more advanced modern varieties replace earlier MVs, but our approach only measures the average health impact across all types of modern varieties adopted.

Third, and most importantly, adoption of MVs often went hand in hand with the spread of other complementary technologies to boost productivity, including fertilizers, irrigation, and pest control (Pingali, 2012), and our estimate of the effect of MV diffusion implicitly includes the effect of adopting these approaches along with MVs. Our findings therefore cannot be read to indicate that MVs should be promoted at the expense of other agricultural technologies. Rather, they speak to the importance of supporting productivity in agriculture as a means of improving lives in developing countries, including the lives of the poor in rural areas. As such, they can inform the recent debate about whether investing in increased smallholder agricultural productivity is an effective strategy for economic development, health improvement, and poverty alleviation in sub-Saharan Africa (Collier and Dercon, 2014, Dercon, 2009). They also suggest that it is reasonable to view with some alarm the steady decline in funding for cereal crop improvement over the last few decades in sub-Saharan Africa, the continent with least diffusion of MVs (Beintema and Stads, 2006, Walker and Alwang, 2015

Some scholars have emphasized potentially negative impacts of the Green Revolution on dietary diversity and a range of environmental outcomes that influence human welfare, arguing that strategic re-evaluation of research and development (R&D) priorities for agriculture is warranted (DeFries et al., 2015, Pingali, 2012, Murgai et al., 2001, Perfecto and Vandermeer, 2010, Brainerd and Menon, 2014). The improved understanding our results provide of welfare impacts of MV adoption can further help to more accurately weigh benefits and drawbacks of agricultural technologies. While recent discussions of malnutrition rightly emphasize the importance of micronutrient supplementation and production (DeFries et al., 2015), our estimates provide compelling evidence that the health benefits of broad-based increases in agricultural productivity should not be overlooked. From the policy perspective, government subsidies for inputs leading to a green revolution as well as investments in extension and R&D programs seem to be important. Even temporary subsidy programs to stimulate green revolution technology adoption may generate high returns, when they have lasting impact on adoption by farmers (Carter et al., 2019).

The health effects of MV diffusion appear to differ substantially based on the sex of the infant, consistent with other evidence of sex-specific effects of income shocks on children (Mulmi et al., 2016, Maccini and Yang, 2009). This gender disparity could reflect both socioeconomic and biological factors. One possibility is that parental discrimination in resource allocation is driving the results. Alternatively, infant males may benefit disproportionately from higher maternal and infant caloric intake due to biological characteristics that contribute to underlying differences in IM rates between the sexes. Identifying which of these mechanisms is at work remains an important avenue for future research.

Our results provide strong evidence for the health benefits of agricultural productivity growth. The substantial decreases in mortality that we observe also likely reflect health improvements among the population of surviving infants, although these gains are less readily observable. Continued investments in agricultural research and development as well as in the diffusion of existing MV varieties may lead to substantial human welfare benefits in areas where MV diffusion (Evenson and Gollin, 2003a, Walker and Alwang, 2015), input intensity (Mueller et al., 2012, Lassaletta et al., 2014), and crop productivity (van Ittersum et al., 2016, van Ittersum et al., 2013, Mueller et al., 2012) remain low. Targeting efforts using new geospatial estimates of malnutrition prevalence (Osgood-Zimmerman et al., 2018) may provide an even larger impact. Further agricultural research will also be needed to minimize the potentially adverse effects of more intensive cultivation on local environmental quality and dietary diversity. These insights will be a key part of ending hunger and raising agricultural productivity and incomes of small-scale food producers.

## Footnotes

1 Undernutrition continues to be recognized as a primary driver of infant mortality (Black et al., 2003). There is a growing interest in investigating the nutritional impacts of agricultural interventions through empirical field studies. Most relevant studies on this question do not measure impacts on health or mortality, but on nutritional outcomes. Moreover, they mostly analyze interventions focused on the diversification of cultivation rather than on increases in the yield of staple crops. Several reviews of the hundreds of papers written on the topic have found little evidence for meaningful impacts (Webb (2013), and citations therein). However, these reviews also warn that the absence of evidence may well reflect the quality of the research on the topic rather than necessarily indicating that such interventions have little impacts.

2 We show in Table A1 that estimates of the association between MV diffusion and infant mortality using country-level data are noisy and do not lead to a conclusive result.

3 Gollin et al. (2018) also adopt a Bartik-style approach using country-level data, but there are two notable distinctions. Firstly, the levels of analysis (country-level versus household) are different, as are the treatments of interest (yields versus MV adoption per se). Because we use micro data, we can also make within-country comparisons, for instance, to analyze heterogeneity by location and household characteristics. In this sense, the two papers provide complementary evidence on welfare implications of HYV adoption. Secondly, our use of grid cell-level predicted MV diffusion and child-level mortality data allows us to control flexibly for potential challenges to causal interpretation of results.

4 Several single-country studies show that agricultural technology improvements are associated with reductions in the likelihood of households living below the poverty line in Mexico, Ethiopia, Rwanda and Uganda (Becerril and Abdulai, 2010, Zeng et al., 2015, Larochelle et al., 2015).

5 In DHS surveys, clusters are usually villages in rural areas and city blocks in urban areas.

6 The dataset is available at http://www.earthstat.org.

7 The dataset also includes soybean, but no EGMV data is available for this crop.

8 The correlation between MVDI based on EarthStat 2000 and SPAM is 0.89; between MVDI based on EarthStat 2000 and EarthStat 1961-65 is 0.88; and between MVDI based on SPAM and EarthStat 1961-65 is 0.83.

9 A particularly relevant recent example is McGuirk and Burke (in press), which examines the reduced form effects of local food prices on conflict. They construct local producer price indices as their explanatory variable by multiplying local crop shares with global time-varying crop prices.

10 We assign MVDI to the year in which the child is born, consistent with the conventional practice in the literature. There will be some misassignment of deaths that occurred in the following year. However, the scope for error is significantly reduced since the time-varying component of MVDI varies in 5-year steps.

11 Preferential allocation of resources to children within a family may depend on the gender as well as the birth order (Jayachandran and Pande, 2017).
