# Supplementary material for: Health Impacts of the Green Revolution: Evidence from 600,000 births across the Developing World
Source: J Health Econ. 2020 Dec;74:102373. doi: 10.1016/j.jhealeco.2020.102373 (PMC7695682; doi:10.1016/j.jhealeco.2020.102373)
Supplement: Supplementary file 1 [file mmc1.pdf]

5 Appendix (Online only)

Figure A1: Distribution of child birth years in main results sample

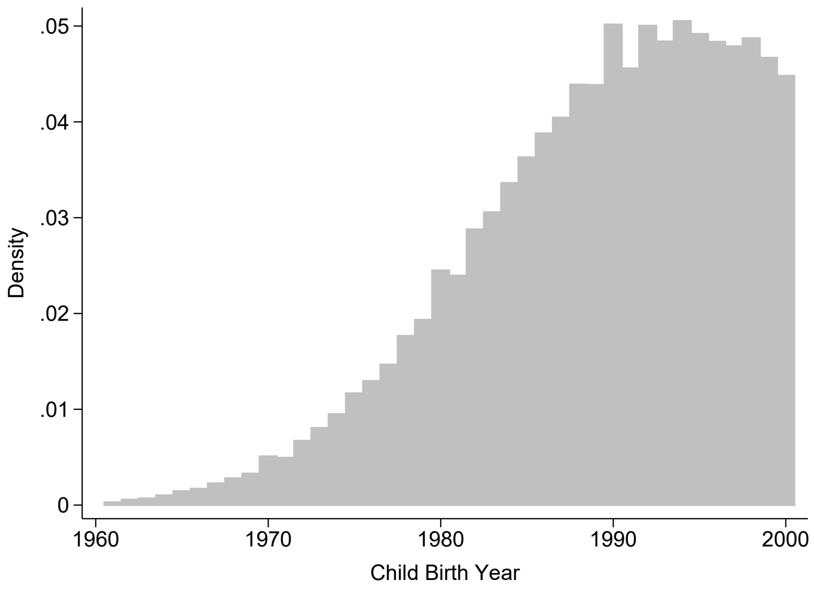

Note: The sample is restricted to rural DHS clusters and mothers who report to have never migrated.

Figure A2: Constructing the MV diffusion indicator for Nigeria using SPAM 2000 cropland areas

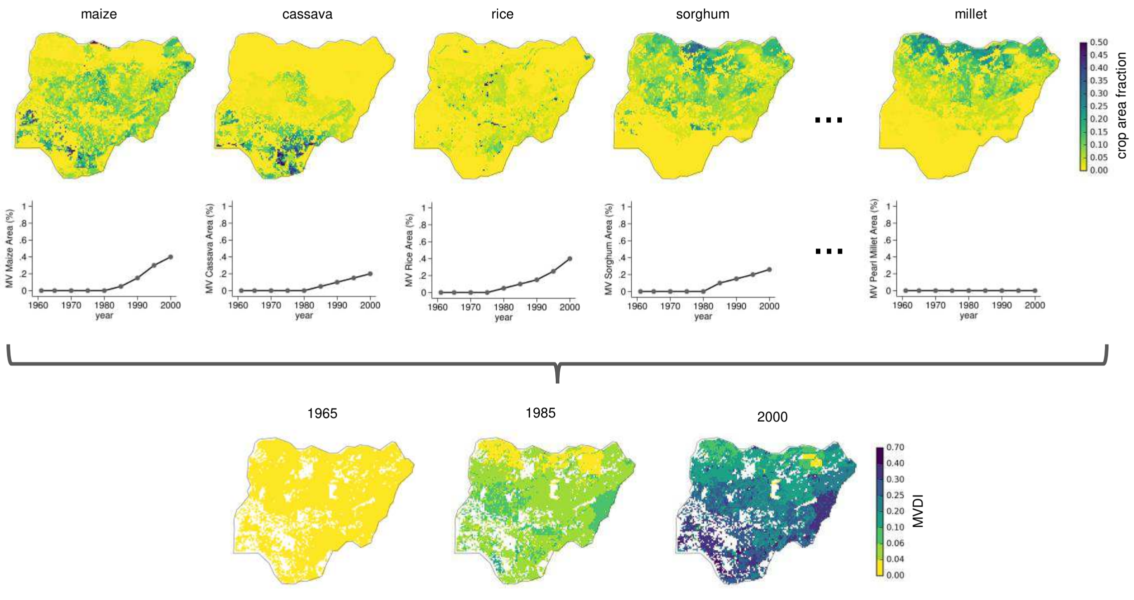

Note: SPAM 2000 dataset from You et al. (2014) includes 10 crops, 5 of which are shown here.

Figure A3: Constructing the MV diffusion indicator for Nigeria using EarthStat cropland areas for 1961-1965

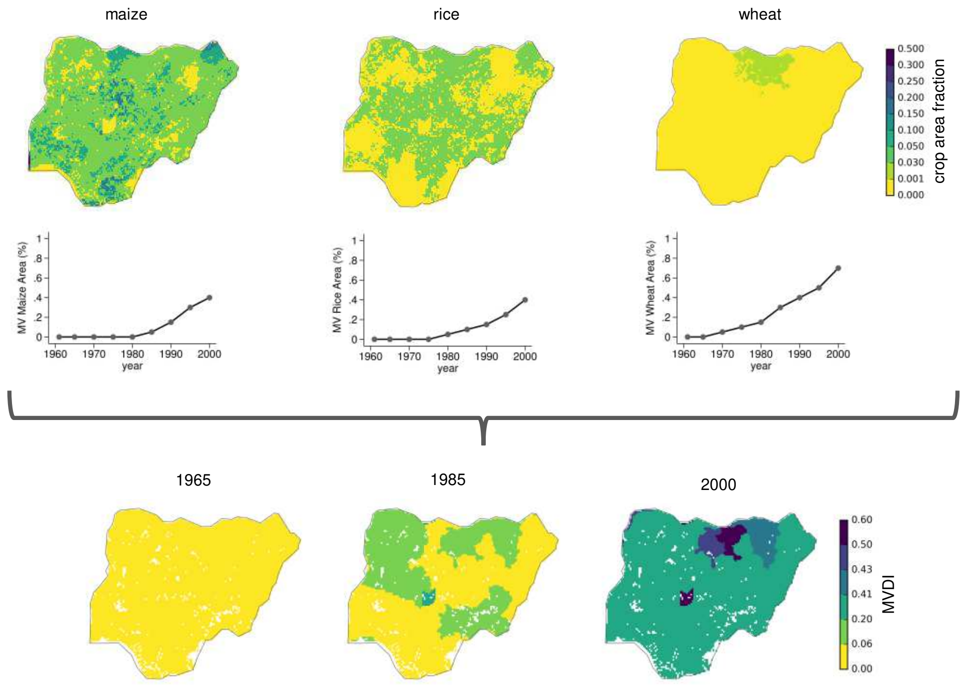

Note: The historical Earthstat dataset from Ray et al. (2012) only includes three crops, and all are shown here.

Figure A4: Distribution of modern varieties in 2000 in India, by relative crop share

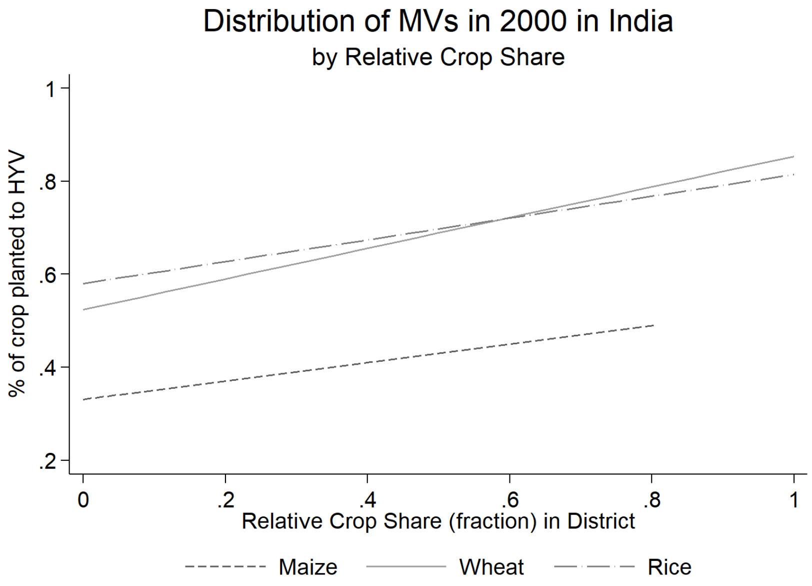

Note: Figure A4 shows the linear fit across districts in India of area planted to MVs in the year 2000 for three crops (maize, wheat and rice) and the area planted of the respective crop as a share of area planted to the three crops. The crop area corresponds to the year 1966, the first year for which data is available ICRISAT (2013).

Figure A5: Randomization tests

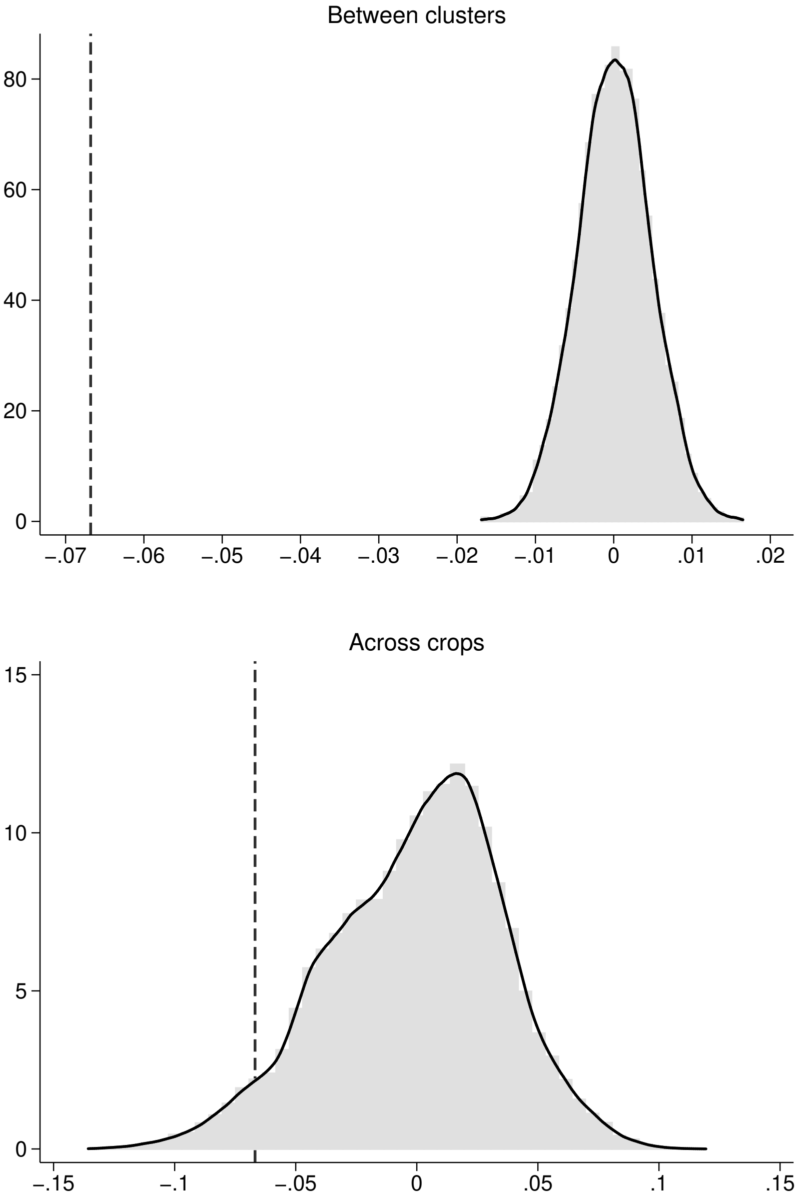

Note: Figure A5 shows distribution of γ estimates after (a) shuffling MVDI across villages while preserving the temporal order within each country (top panel), and (b) random assignment of EGMV across crops, independently within each country, before construction of MVDI (bottom panel). The estimate γ is derived from: $y_{ivct} = \gamma MVDI_{vct}^{placebo} + u_v + Z_{ct} + X_{ivct} + e_{ivct}$ where y_{ivct} is a binary indicator of infant mortality i.e. whether child i born in year t in DHS sampling cluster v in country c died in the first year of life; u_v are cluster fixed effects and Z_{ct} are country-by-year FE; X_{ivct} includes quadratic in mother's age (at birth of child) and sex of child; and e_{ivct} are idiosyncratic errors clustered at subnational (admin) level. The sample is restricted to rural DHS clusters and mothers who report to have never migrated. The distributions reflect 10,000 randomization draws, and vertical line shows actual point estimate of γ from Table 2 using EarthStat 1961-1965 cropped area dataset. The p-value is $<$ 0.001 and $<$ 0.05.

Figure A6: Estimated impact of modern varieties on infant mortality across specifications

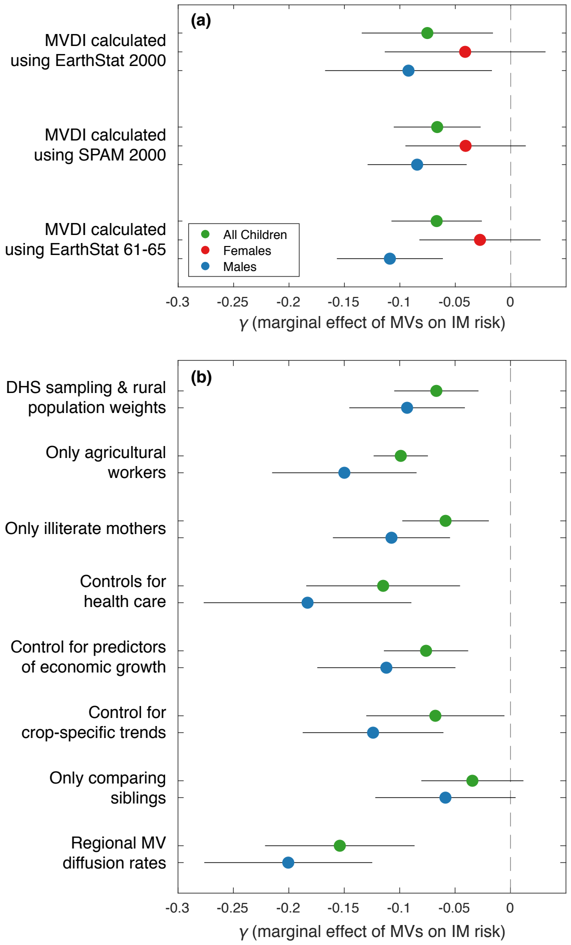

Note: Each estimate in Figure A6 represents γ from the following estimating equation: $y_{ivct} = \gamma MVDI_{vct} + u_v + Z_{ct} + X_{ivct} + e_{ivct}$ where y_{ivct} is a binary indicator of infant mortality i.e. whether child i born in year t in DHS sampling cluster v in country c died in the first year of life; u_v are cluster fixed effects and Z_{ct} are country-by-year FE; X_{ivct} includes quadratic in mother's age (at birth of child) and sex of child; and e_{ivct} are idiosyncratic errors clustered at subnational (admin) level. 95% confidence intervals shown. The sample is restricted to rural DHS clusters and mothers who report to have never migrated. Panel (a) shows estimates using three different crop maps to construct MVDI and reports estimates by child sex for each crop map. Panel (b) reports estimates for both sexes and for males from the following variants on the model: weighting observations by the DHS sampling weights multiplied by the country's rural population divided by the rural sample size for the country across all DHS survey rounds; limiting the sample to mothers that report being agricultural wage laborers; limiting to mothers who are illiterate; controlling for mother's antenatal care visits, duration of breastfeeding, and child vaccination; detrending the data as a function of distance to coast; detrending the data according to crop mix; only comparing siblings by adding mother fixed effects; and constructing the MVDI using average MV diffusion in the country's region within the continent, without including the country's MV in the average.

Table A1: Country-level associations between MV diffusion and infant mortality

	(1)	(2)	(3)	(4)
Panel A: All Countries				
MV (11 crops)	52.99		10.80	
	(18.36)***		(40.04)	
MV (cereals)		29.06		-31.20
		(13.78)**		(34.71)
N	700	700	700	700
Countries	86	86	86	86
Panel B: Geocoded DHS Countries				
MV (11 crops)	7.51		65.41	
	(26.54)		(73.59)	
MV (cereals)		-11.89		12.90
		(17.65)		(62.92)
N	305	305	305	305
Countries	37	37	37	37
Region $\times$ year FE	Yes	Yes	No	No
Country specific trends	No	No	Yes	Yes

Note: Table A1 presents results for the following estimating equation: $y_{ct} = \gamma MV_{ct} + u_c + f(t) + e_{ct}$ where y_{ct} is the infant mortality in country c at time t (number of infants dying per 1,000 births); MV_{ct} is the crop area weighted MV adoption in country c at time t for 11 crops (barley, cassava, groundnut, lentil, maize, bean, millet, potato, rice, sorghum and wheat) or 5 cereals (maize, millet, rice, sorghum and wheat) Evenson and Gollin (2003b); u_c are country fixed effects and $f(t)$ are region-by-year fixed effects or country-specific linear time trends; and e_{ct} is the idiosyncratic error term that is clustered at country level. Standard errors in parentheses, * p<0.10, ** p<0.05, *** p<0.01

Table A2: Number of surveys and infants in union of estimating samples, by country

Country	Surveys	Females	Males	Total
EAP-Cambodia	2 (2000, 2005)	17,031	17,786	34,817
EAP-Philippines	2 (2003, 2008)	3,771	4,151	7,922
LAC-Bolivia	1 (2000)	2,828	2,889	5,717
LAC-Colombia	1 (2000)	3,638	3,765	7,403
LAC-Dominican Rep.	1 (2007)	6,339	6,758	13,097
LAC-Haiti	2 (2000, 2006)	8,694	9,026	17,720
LAC-Peru	2 (2000, 2005)	16,387	17,157	33,544
NA-Egypt	6 (1992, 1995, 2000, 2003, 2005, 2008)	56,143	60,058	116,201
NA-Morocco	1 (2004)	3,143	3,370	6,513
SA-Bangladesh	3 (2000, 2004, 2007)	4,745	4,891	9,636
SA-India	1 (2016)	12,316	14,092	26,408
SA-Nepal	2 (2001, 2006)	2,633	2,731	5,364
SSA-Benin	2 (1996, 2001)	5,494	5,841	11,335
SSA-Burkina Faso	3 (1993, 1999, 2003)	11,252	11,910	23,162
SSA-Central African Republic	1 (1995)	2,367	2,445	4,812
SSA-Cote d'Ivoire	1 (1994)	2,199	2,181	4,380
SSA-Comoros	2 (1991, 2004)	2,729	2,806	5,535
SSA-Congo, Dem. Rep.	1 (2007)	1,841	2,036	3,877
SSA-Ethiopia	2 (2000, 2005)	17,375	18,758	36,133
SSA-Ghana	4 (1993, 1998, 2003, 2008)	4,981	5,286	10,267
SSA-Guinea	1 (2005)	5,012	5,399	10,411
SSA-Kenya	2 (2003, 2009)	2,632	2,895	5,527
SSA-Liberia	2 (2007, 2009)	2,135	2,338	4,473
SSA-Mali	3 (1996, 2001, 2006)	18,458	19,298	37,756
SSA-Malawi	3 (2000, 2004, 2010)	22,869	23,319	46,188
SSA-Namibia	2 (2000, 2007)	2,842	2,792	5,634
SSA-Niger	2 (1992, 1998)	8,544	8,917	17,461
SSA-Nigeria	3 (1990, 2003, 2008)	17,718	18,704	36,422
SSA-Rwanda	1 (2005)	1,941	1,938	3,879
SSA-Senegal	4 (1993, 1997, 2005, 2009)	13,715	14,321	28,036
SSA-Sierra Leone	1 (2008)	1,504	1,647	3,151
SSA-Swaziland	1 (2007)	538	506	1,044
SSA-Togo	2 (1988, 1998)	3,324	3,427	6,751
SSA-Tanzania	2 (1999, 2008)	2,752	2,702	5,454
SSA-Uganda	2 (2001, 2006)	3,086	3,114	6,200
SSA-Zambia	1 (2007)	1,456	1,395	2,851
SSA-Zimbabwe	2 (1999, 2006)	3,440	3,510	6,950
Total	74	297,872	314,159	612,031

Note: EAP refers to East Asia and Pacific, LAC refers to Latin America and the Caribbean, NA refers to North Africa, SA refers to South Asia, and SSA refers to sub-Saharan Africa.

Table A3: Validation of MVDI using subnational data from India

	(1) EarthStat (circa 2000)	(2) EarthStat (1961-1965)
Panel A:		
MVDI	0.2978	0.4272
	(0.0954)***	(0.1130)***
N	2,408	2,408
Panel B:		
Adjusted MVDI	0.4060	0.4665
	(0.0888)***	(0.1120)***
N	2,408	2,408

Note: Table A3 presents estimates of the regression: $MV_{dt} = \beta MVDI_{dt} + u_d + v_t + e_{dt}$ where, MV_{dt} is the area-weighted adoption of modern varieties in district d at time t (constructed using district-level data (ICRISAT, 2013)); $MVDI_{dt}$ refers to either the constructed MVDI variable using Equation 1 in district d at time t (panel A), or the adjusted MVDI which constructs MVDI such that higher modern variety diffusion Evenson and Gollin (2003a) is assigned to districts growing relatively larger shares of the crop (panel B); u_d are district fixed effects and v_t are year fixed effects; and e_{dt} is the idiosyncratic error term that is clustered at district level. MVDI using EarthStat 2000 areas is only calculated using the five crops available in the Indian administrative data [i.e. $j = 5$ (rice, wheat, maize, sorghum, millet)]. The definition of MVDI based on EarthStat 1961-1965 areas uses $j = 3$ (rice, wheat, maize) and is unchanged. Standard errors in parentheses, * p<0.10, ** p<0.05, *** p<0.01.

Table A4: Impact of modern variety diffusion on sex ratio

	(1) EarthStat (circa 2000)	(2) SPAM (circa 2000)	(3) EarthStat (1961-1965)
MVDI	0.0760	0.0217	-0.0240
	(0.0348)**	(0.0279)	(0.0276)
N	597,247	577,101	581,490
Mean	.51	.51	.51

Note: Each estimate in Table A4 represents γ from the following estimating equation: $y_{ivct} = \gamma MVDI_{vct} + u_v + Z_{ct} + X_{ivct} + e_{ivct}$ where y_{ivct} is a binary indicator of whether child in DHS cluster v in country c born in year t is a boy; u_v are cluster fixed effects and Z_{ct} are country-by-year fixed effects; X_{ivct} includes quadratic in mother's age (at birth of child); and e_{ivct} are idiosyncratic errors clustered at subnational (admin) level. The sample is restricted to rural DHS clusters and mothers who report to have never migrated. Standard errors in parentheses, * p<0.10, ** p<0.05, *** p<0.01.

Table A5: Impact of modern varieties on infant mortality (within parity and within mother estimations)

	(1)	(2)
Panel A: All Children		
MVDI	-0.0675	-0.0344
	(0.0211)***	(0.0235)
N	581,490	557,043
Mean	.1	.1
Panel B: Females		
MVDI	-0.0283	0.0182
	(0.0280)	(0.0396)
N	281,724	240,757
Mean	.097	.1
Panel C: Males		
MVDI	-0.1101	-0.0587
	(0.0248)***	(0.0323)*
N	297,236	257,047
Mean	.11	.11
Controls	Birth order FE	Mother FE

Note: Table A5, column 1 represents γ from the following estimating equation: $y_{ivct} = \gamma MVDI_{vct} + u_v + w_o + Z_{ct} + X_{ivct} + e_{ivct}$ where y_{ivct} is a binary indicator of infant mortality i.e. whether child i born in year t in DHS sampling cluster v in country c died in the first year of life; u_v are cluster fixed effects; w_o are birth order fixed effects (so that only children of the same parity are compared) and Z_{ct} are country-by-year fixed effects; X_{ivct} includes quadratic in mother's age (at birth of child) and sex of child; and e_{ivct} are idiosyncratic errors clustered at subnational (admin) level. Column 2 represents γ from the following estimating equation: $y_{imvct} = \gamma MVDI_{vct} + u_m + Z_{ct} + X_{ivct} + e_{ivct}$ where all the terms are same as defined earlier with two exceptions. First, the m subscript has been added to emphasize that child i belongs to mother m. Additionally, u_m refers to mother fixed effects (so that only children born to the same mother are being compared). MVDI is calculated using the EarthStat 1961-1965 crop map data. The sample is only restricted to rural DHS clusters and mothers who have never migrated. Standard errors in parentheses, * p<0.10, ** p<0.05, *** p<0.01.

Table A6: Heterogenous impacts of modern varieties on infant mortality, by distance to cities

	(1)<br>Distance to 500k population cities	(2)<br>Distance to 1m population cities
Panel A: All Children		
MVDI	-0.0546	-0.0335
	(0.0248)*	(0.0199)
MVDI × Distance	-0.0048	-0.0115
	(0.0042)	(0.0041)**
N	580,426	580,426
Panel B: Females		
MVDI	-0.0354	-0.0060
	(0.0284)	(0.0221)
MVDI × Distance	0.0031	-0.0075
	(0.0050)	(0.0048)
N	281,271	281,271
Panel C: Males		
MVDI	-0.0756	-0.0632
	(0.0337)*	(0.0308)*
MVDI × Distance	-0.0130	-0.0157
	(0.0063)*	(0.0053)**
N	296,628	296,628

Note: Each estimate in Table A6 represents γ and θ from the following estimating equation run for different distances separately: $y_{ivct} = \gamma MVDI_{vct} + \theta MVDI_{vct} \times W_{vc} + u_v + Z_{ct} + X_{ivct} + e_{ivct}$ where y_{ivct} is a binary indicator of infant mortality i.e. whether child i born in year t in DHS sampling cluster v in country c died in the first year of life; W_{vc} is the time-invariant distance of cluster v in country c from cities of different population size, either cities with population more than 500,000 (column 1) or distance to cities with population more than 1,000,000 (column 2); u_v are cluster fixed effects and Z_{ct} are country-by-year fixed effects; X_{ivct} includes quadratic in mother's age (at birth of child) and sex of child; and e_{ivct} are idiosyncratic errors clustered at subnational (admin) level. MVDI is calculated using the EarthStat 1961-1965 crop map data. Distance is measured in hundreds of kilometers. Standard errors in parentheses, * p<0.10, ** p<0.05, *** p<0.01.

Table A7: Impact of modern varieties on infant mortality, weighting observations

	(1)<br>EarthStat<br>(circa 2000)	(2)<br>SPAM<br>(circa 2000)	(3)<br>EarthStat<br>(1961-1965)
Panel A: All Children			
MVDI	-0.0837	-0.0374	-0.0669
	(0.0319)***	(0.0393)	(0.0194)***
N	597,247	577,101	581,490
Mean	.097	.097	.097
Panel B: Females			
MVDI	-0.0387	-0.0195	-0.0299
	(0.0348)	(0.0448)	(0.0246)
N	289,183	279,563	281,724
Mean	.092	.091	.092
Panel C: Males			
MVDI	-0.1080	-0.0414	-0.0933
	(0.0454)**	(0.0526)	(0.0266)***
N	305,379	295,014	297,236
Mean	.1	.1	.1

Note: Each estimate in Table A7 represents γ from the following estimating equation: $y_{ivct} = \gamma MVDI_{vct} + u_v + Z_{ct} + X_{ivct} + e_{ivct}$ where y_{ivct} is a binary indicator of infant mortality i.e. whether child i born in year t in DHS sampling cluster v in country c died in the first year of life; u_v are cluster fixed effects and Z_{ct} are country-by-year FE; X_{ivct} includes quadratic in mother's age (at birth of child) and sex of child; and e_{ivct} are idiosyncratic errors. Columns report estimates obtained through the use of the three global crop map datasets. The sample is restricted to rural DHS clusters and mothers who report to have never migrated. Observations are weighted using the DHS sampling weights multiplied by the country's rural population divided by the sample size for that country (across all DHS surveys) in the regression. Since we are using a subsample of the DHS (rural mothers who have never migrated), DHS sampling weights in our sample are re-normalized to mean 1. Standard errors in parentheses are clustered at the subnational (admin) level. Standard errors in parentheses, * p<0.10, ** p<0.05, *** p<0.01.

Table A8: Association between MVDI and maternal, neonatal and child health interventions at the DHS cluster level

	(1) EarthStat (circa 2000)	(2) SPAM (circa 2000)	(3) EarthStat (1961-1965)
Panel A: Access to health care			
MVDI	0.0244	0.0091	0.1835
	(0.1221)	(0.0685)	(0.1328)
N	1,770	1,749	1,755
Mean	.44	.43	.43
Panel B: ANC visits			
MVDI	0.8718	0.3201	0.0984
	(0.4503)*	(0.2896)	(0.3325)
N	6,050	5,848	5,903
Mean	2.3	2.3	2.3
Panel C: Institutional delivery			
MVDI	0.0342	0.0158	-0.1789
	(0.1056)	(0.0587)	(0.0752)**
N	6,056	5,856	5,909
Mean	.27	.27	.27
Panel D: Breastfeeding			
MVDI	-0.0698	-0.0321	0.0511
	(0.0511)	(0.0246)	(0.0397)
N	6,059	5,860	5,912
Mean	.32	.32	.32
Panel E: Vaccination			
MVDI	-0.0091	0.0309	-0.0356
	(0.0557)	(0.0219)	(0.0505)
N	5,878	5,683	5,774
Mean	.81	.81	.81

Note: Table A8 presents results for the following estimating equation: $H_{vct} = \gamma MVDI_{vct} + u_c + Z_{ct} + e_{vct}$ where H_{vct} is a measure of Maternal, Neonatal and Child Interventions (MNCH) in DHS cluster *v* in country *c* in survey year *t*; u_c are country fixed effects; Z_{ct} are country $\times$ year fixed effects; and e_{ivct} are idiosyncratic errors clustered at subnational (admin) level. In Panel A, access to health care is determined by the fraction of women who reported that distance was not an obstacle in the use of medical care; in panel B, antenatal care is defined as the average number of antenatal visits reported by women; in panel C, institutional delivery was defined as fraction of children who were reported to have been delivered in any kind of health facility; in panel D, breastfeeding is calculated as the fraction of women who reported to be breastfeeding at the time of survey; and in panel E, vaccination rates are calculated as the fraction of children who received any vaccination (BCG, TB, DPT, Polio, Measles, etc.). The estimating sample consists only of rural DHS clusters and the proportions are always calculating after restricting sample to mothers who reported to have never migrated. Standard errors in parentheses, * p<0.10, ** p<0.05, *** p<0.01.

Table A9: Impact of modern varieties on infant mortality, controlling for subnational geographic trends

	(1)	(2)	(3)	(4)
Panel A: All Children				
MVDI	-0.0836	-0.0762	-0.0470	-0.0621
	(0.0236)***	(0.0194)***	(0.0185)***	(0.0270)**
N	579,797	580,426	581,490	581,490
Mean	.1	.1	.1	.1
Panel B: Females				
MVDI	-0.0616	-0.0404	-0.0232	-0.0098
	(0.0297)**	(0.0273)	(0.0246)	(0.0379)
N	280,410	281,271	281,724	281,724
Mean	.097	.097	.097	.097
Panel C: Males				
MVDI	-0.1232	-0.1120	-0.0944	-0.1037
	(0.0339)***	(0.0244)***	(0.0243)**	(0.0327)***
N	295,814	296,628	297,236	297,236
Mean	.11	.11	.11	.11
Fixed Effects	Admin1 x Year	Geography	Crop area I	Crop area II

Note: Table A9 reports γ from the following estimating equation: $y_{ivct} = \gamma MVDI_{vct} + u_v + Z_{ct} + X_{ivct} + f(t) + e_{ivct}$ where y_{ivct} is a binary indicator of infant mortality i.e. whether child i born in year t in DHS sampling cluster v in country c died in the first year of life; u_v are cluster fixed effects; Z_{ct} are country-by-year fixed effects; $f(t)$ refer to additional subnational fixed effects (defined below); X_{ivct} includes quadratic in mother's age (at birth of child) and sex of child; and e_{ivct} are idiosyncratic errors clustered at subnational (admin) level. Column (1) adds Admin1 $\times$ year FE; column (2) adds $A_{ct} \times D^{Coast}_{vc} + B_{ct} \times D^{Cities}_{vc}$ where (A_{ct}, B_{ct}) and the distance of each cluster from the coast (D^{Coast}) and from cities (D^{Cities}); column (3) add crop area $\times$ year FE and crop area $\times$ country FE; and column (4) adds crop-specific country year fixed effects i.e. $\sum_j \alpha_j A^{(j)}_{ct} \times CropArea_{jvc}$ where $A^{(j)}_{ct}$ refers to the cropped area of each crop j in the location in question, for the three crops in the EarthStat 1961-1965 crop map data (maize, rice and wheat). MVDI is calculated using the EarthStat 1961-1965 crop maps. The sample is restricted to rural DHS clusters and mothers who have never migrated. Standard errors in parentheses, * p<0.10, ** p<0.05, *** p<0.01.

Table A10: Impact of modern variety diffusion on infant mortality with alternative within-country MV distribution assumptions

	(1) EarthStat (1961-1965) (Equation 1)	(2) EarthStat (1961-1965) (Adjusted)
Panel A: All Children		
MVDI	-0.0668	-0.0700
	(0.0208)***	(0.0209)***
N	581,490	581,490
Mean	.1	.1
Panel B: Girls		
MVDI	-0.0277	-0.0371
	(0.0279)	(0.0291)
N	281,724	281,724
Mean	.097	.097
Panel C: Boys		
MVDI	-0.1090	-0.1040
	(0.0244)***	(0.0241)***
N	297,236	297,236
Mean	.11	.11

Note: Each estimate in Table A10 represents γ from the following estimating equation: $y_{ivct} = \gamma MVDI_{vct} + u_v + Z_{ct} + X_{ivct} + e_{ivct}$ where y_{ivct} is a binary indicator of infant mortality i.e. whether child i born in year t in DHS sampling cluster v in country c died in the first year of life; u_v are cluster fixed effects and Z_{ct} are country-by-year FE; X_{ivct} includes quadratic in mother's age (at birth of child) and sex of child; and e_{ivct} are idiosyncratic errors clustered at subnational (admin) level. 95% confidence intervals shown. The sample is restricted to rural DHS clusters and mothers who report to have never migrated. MVDI in (1) assumes that all areas of a country growing a particular crop receive the respective national EGMV, whereas the Adjusted MVDI in (2) assumes that relatively more EGMV went to parts of a country cultivating relatively more of the respective crop. Standard errors in parentheses, * p<0.10, ** p<0.05, *** p<0.01.

Table A11: Impact of modern varieties on infant mortality in subsamples following MV arrival

	(1)<br>MV>0	(2)<br>MV>5%	(3)<br>MV>10%
Panel A:			
Within 10 years of MV arrival			
MVDI	-0.0381	-0.0264	-0.0678
	(0.0425)	(0.0386)	(0.0393)*
N	105,825	123,266	130,718
Panel B:			
Within 15 years of MV arrival			
MVDI	-0.0637	-0.0683	-0.1017
	(0.0304)**	(0.0286)**	(0.0290)***
N	160,720	174,592	176,503

Note: Each estimate in Table A11 represents γ from the following estimating equation: $y_{ivct} = \gamma MVDI_{vct} + u_v + Z_{ct} + X_{ivct} + e_{ivct}$ where y_{ivct} is a binary indicator of infant mortality i.e. whether child i born in year t in DHS sampling cluster v in country c died in the first year of life; u_v are cluster fixed effects and Z_{ct} are country-by-year fixed effects; X_{ivct} includes quadratic in mother's age (at birth of child); and e_{ivct} are idiosyncratic errors clustered at subnational (admin) level. Columns report estimates obtained using EarthStat 1961-65 crop map. The sample is only restricted to boys born in rural DHS clusters to mothers who have never migrated and with $k \in \{10, 15\}$ years of MV arrival. Standard errors in parentheses, * p<0.10, ** p<0.05, *** p<0.01.

Table A12: Impact of modern varieties on infant mortality, including migrants

	(1)<br>EarthStat<br>(circa 2000)	(2)<br>SPAM<br>(circa 2000)	(3)<br>EarthStat<br>(1961-1965)
Panel A: All Children			
MVDI	-0.0030	-0.0146	-0.0405
	(0.0184)	(0.0119)	(0.0131)***
N	1793575	1720349	1747701
Mean	.094	.094	.095
Panel B: Females			
MVDI	0.0109	-0.0024	-0.0303
	(0.0208)	(0.0147)	(0.0150)**
N	867,543	832,442	845,492
Mean	.088	.088	.088
Panel C: Males			
MVDI	-0.0133	-0.0272	-0.0527
	(0.0223)	(0.0145)*	(0.0170)***
N	925,198	887,120	901,421
Mean	.1	.1	.1

Note: Each estimate in Table A12 represents γ from the following estimating equation: $y_{ivct} = \gamma MVDI_{vct} + u_v + Z_{ct} + X_{ivct} + e_{ivct}$ where y_{ivct} is a binary indicator of infant mortality i.e. whether child i born in year t in DHS sampling cluster v in country c died in the first year of life; u_v are cluster fixed effects and Z_{ct} are country-by-year fixed effects; X_{ivct} includes quadratic in mother's age (at birth of child) and sex of child; and e_{ivct} are idiosyncratic errors clustered at subnational (admin) level. Columns report estimates obtained through the use of the three global crop maps. The sample is only restricted to rural DHS clusters; the estimating sample includes all mothers, both migrants and never movers. Standard errors in parentheses, * p<0.10, ** p<0.05, *** p<0.01.

Table A13: Impact of modern varieties on infant mortality, urban vs. rural locations

	(1)<br>Rural DHS clusters	(2)<br>Urban DHS clusters
Panel A: All Children		
MVDI	-0.0668	-0.0238
	(0.0208)***	(0.0337)
N	581,490	170,392
Mean	.1	.066
Panel B: Females		
MVDI	-0.0277	0.0114
	(0.0279)	(0.0386)
N	281,724	81,931
Mean	.097	.06
Panel C: Males		
MVDI	-0.1090	-0.0572
	(0.0244)***	(0.0412)
N	297,236	85,782
Mean	.11	.074

Note: Each estimate in Table A13 represents γ from the following estimating equation: $y_{ivct} = \gamma MVDI_{vct} + u_v + Z_{ct} + X_{ivct} + e_{ivct}$ where y_{ivct} is a binary indicator of infant mortality i.e. whether child i born in year t in DHS sampling cluster v in country c died in the first year of life; $MVDI_{vct}$ refers to MV diffusion in cluster v in country c at time t, derived from the EarthStat 1961-1965 crop map; u_v are cluster fixed effects and Z_{ct} are country-by-year fixed effects; X_{ivct} includes quadratic in mother's age (at birth of child) and sex of child; and e_{ivct} are idiosyncratic errors clustered at subnational (admin) level. Columns 1 and 2 report estimates obtained from running the regression seperately in rural and urban areas. The sample is restricted to never movers. Standard errors in parentheses, * p<0.10, ** p<0.05, *** p<0.01.

Table A14: Impact of modern varieties on infant mortality after accounting for recall bias

	(1) EarthStat (circa 2000)	(2) SPAM (circa 2000)	(3) EarthStat (1961-1965)
Panel A: Births $\geq$ 1980s			
MVDI	-0.0740	-0.0596	-0.0547
	(0.0345)**	(0.0219)***	(0.0276)**
N	522,276	504,983	507,924
Mean	.095	.094	.096
Panel B: Young mothers			
MVDI	-0.0976	-0.0644	-0.0865
	(0.0511)*	(0.0300)**	(0.0409)**
N	363,558	350,176	354,528
Mean	.1	.1	.1
Panel C: Literate mothers			
MVDI	-0.0057	-0.0786	-0.0768
	(0.0541)	(0.0311)**	(0.0476)
N	137,075	134,699	132,963
Mean	.072	.073	.073
Panel D: Recall $\leq$ 20 years			
MVDI	-0.1088	-0.0732	-0.0933
	(0.0404)***	(0.0233)***	(0.0387)**
N	485,534	468,950	473,632
Mean	.097	.096	.098
Panel E: Control for recall year			
MVDI	-0.0752	-0.0663	-0.0668
	(0.0302)**	(0.0200)***	(0.0208)***
N	597,247	577,101	581,490
Mean	.1	.1	.1
Panel F: Recall fixed effects			
MVDI	-0.0747	-0.0668	-0.0658
	(0.0299)**	(0.0201)***	(0.0209)***
N	597,247	577,101	581,490
Mean	.1	.1	.1
Panel G: Inverse recall weights			
MVDI	-0.0837	-0.0694	-0.0664
	(0.0334)**	(0.0238)***	(0.0289)***
N	597,247	577,101	581,490
Mean	.1	.1	.1

Note: Each estimate in Table A14 represents γ from the following estimating equation: $y_{ivct} = \gamma MVDI_{vct} + u_v + Z_{ct} + X_{ivct} + e_{ivct}$ where y_{ivct} is a binary indicator of infant mortality i.e. whether child i born in year t in DHS sampling cluster v in country c died in the first year of life; u_v are cluster fixed effects and Z_{ct} are country-by-year fixed effects; X_{ivct} includes quadratic in mother's age (at birth of child) and sex of child; and e_{ivct} are idiosyncratic errors clustered at sub-national (admin) level. Panel A restricts the estimating sample to birth after 1980s (dropping 1960s and 1970s); panel B restricts sample to mothers in the age group 15-40 years at the time of survey; panel C restricts the sample to mother who are literate; panel D restricts the sample to births that are within 20 years of survey year; panel E adds controls for the distance between the birth year and survey year; panel F adds a fixed effect for each recall year; and panel G weighs the regression by the inverse of the recall period to reduce the importance of older births. Columns report estimates obtained through the use of the three global crop maps. The sample is further restricted to rural DHS clusters and mothers who report to have never migrated. Standard errors in parentheses, * p<0.10, ** p<0.05, *** p<0.01.

Table A15: Test for pre-trends

	(1)
Future MVDI (t+5)	0.0006
	(0.0020)
N	446,151

Note: Table A15 presents the result from regressing residuals from the main estimating equation in Table 2 (using EarthStat 1961-1965 crop map data) on MVDI in the next time period. Since the MV data is observed quinquennially, future MVDI is defined as MVDI after 5 years after child's birth. Standard errors in parentheses, * $p<0.10$, ** $p<0.05$, *** $p<0.01$.
